# The nature of spin excitations in the one-third magnetization plateau phase of Ba_3_CoSb_2_O_9_

**DOI:** 10.1038/s41467-018-04914-1

**Published:** 2018-07-10

**Authors:** Y. Kamiya, L. Ge, Tao Hong, Y. Qiu, D. L. Quintero-Castro, Z. Lu, H. B. Cao, M. Matsuda, E. S. Choi, C. D. Batista, M. Mourigal, H. D. Zhou, J. Ma

**Affiliations:** 1 Condensed Matter Theory Laboratory, RIKEN, Wako, Saitama, 351-0198 Japan; 2 School of Physics, Georgia Institute of Technology, Atlanta, GA 30332 USA; 3 Neutron Scattering Division, Oak Ridge National Laboratory, Oak Ridge, TN 37831 USA; 4 NIST Centre for Neutron Research, National Institute of Standards and Technology, Gaithersburg, MD 20899 USA; 5 Helmholtz-Zentrum Berlin für Materialien und Energie, D-14109 Berlin, Germany; 6 National High Magnetic Field Laboratory, Florida State University, Tallahassee, FL 32310 USA; 7 Department of Physics and Astronomy, University of Tennessee, Knoxville, TN 37996 USA; 8 Neutron Scattering Division and Shull-Wollan Center, Oak Ridge National Laboratory, Oak Ridge, TN 37831 USA; 9 Key Laboratory of Artificial Structures and Quantum Control, Department of Physics and Astronomy, Shanghai Jiao Tong University, 200240 Shanghai, China; 10 Collaborative Innovation Center of Advanced Microstructures, 210093 Nanjing, Jiangsu China

## Abstract

Magnetization plateaus in quantum magnets—where bosonic quasiparticles crystallize into emergent spin superlattices—are spectacular yet simple examples of collective quantum phenomena escaping classical description. While magnetization plateaus have been observed in a number of spin-1/2 antiferromagnets, the description of their magnetic excitations remains an open theoretical and experimental challenge. Here, we investigate the dynamical properties of the triangular-lattice spin-1/2 antiferromagnet Ba_3_CoSb_2_O_9_ in its one-third magnetization plateau phase using a combination of nonlinear spin-wave theory and neutron scattering measurements. The agreement between our theoretical treatment and the experimental data demonstrates that magnons behave semiclassically in the plateau in spite of the purely quantum origin of the underlying magnetic structure. This allows for a quantitative determination of Ba_3_CoSb_2_O_9_ exchange parameters. We discuss the implication of our results to the deviations from semiclassical behavior observed in zero-field spin dynamics of the same material and conclude they must have an intrinsic origin.

## Abstract (web summary)

Frustrated magnetic materials attract significant interest because their properties can become dominated by quantum fluctuations. Here the authors show that excitations in the plateau phase of a quantum magnet can be understood semiclassically even though the ground state involves strong quantum effects.

## Introduction

Quantum fluctuations favor collinear spin order in frustrated magnets^1–3^, which can be qualitatively different from the classical limit (*S* → ∞)^4^. In particular, quantum effects can produce magnetization plateaus^5–10^, where the magnetization is pinned at a fraction of its saturation value. Magnetization plateaus can be interpreted as crystalline states of bosonic particles, and are naturally stabilized by easy-axis exchange anisotropy, which acts as strong off-site repulsion^11–14^. However, the situation is less evident and more intriguing for isotropic Heisenberg magnets, which typically have no plateaus in the classical limit. In a seminal work, Chubukov and Golosov predicted the 1/3 magnetization plateau in the quantum triangular lattice Heisenberg antiferromagnet (TLHAFM), corresponding to an up–up–down (UUD) state^5^. Their predictions were confirmed by numerical studies^6,15–21^ and extended to plateaus in other models^6^. Experimentally, the 1/3 plateau has been observed in the spin-1/2 isosceles triangular lattice material Cs_2_CuBr_4_^22–26^, as well as in the equilateral triangular lattice materials RbFe(MoO_4_)_2_ (*S* = 5/2)^27–29^ and Ba_3_CoSb_2_O_9_ (effective *S* = 1/2)^30–39^.

Notwithstanding the progress in the search of quantum plateaus, much less is known about their excitation spectra. Given that they are stabilized by quantum fluctuations, it is natural to ask if these fluctuations strongly affect the excitation spectrum. The qualitative difference between the plateau and the classical orderings may appear to invalidate spin-wave theory. For instance, the UUD state in the equilateral TLHAFM is not a classical ground state unless the magnetic field *H* is fine-tuned^40^. Consequently, a naive spin wave treatment is doomed to instability. On the other hand, spin wave theory builds on the assumption of an ordered moment |〈**S**_**r**_〉| close to the full moment. Given that a sizable reduction of |〈**S**_**r**_〉| is unlikely within the plateau because of the gapped nature of the spectrum, a spin wave description could be adequate. Although this may seem in conflict with the order-by-disorder mechanism^1–3^ stabilizing the plateau^40–42^, this phenomenon is produced by the zero-point energy correction $E_{{\mathrm{zp}}} = (1/2)\mathop {\sum}\nolimits_{\mathbf{q}} \omega _{\mathbf{q}} + O(S^0)$ (*ω*_**q**_ is the spin wave dispersion), which does not necessarily produce a large moment size reduction.

Here, one of our goals is to resolve this seemingly contradictory situation. Recently, Alicea et al. developed a method to fix the unphysical spin-wave instability^40^. This proposal awaits experimental verification because the excitation spectrum has not been measured over the entire Brillouin zone for any fluctuation-induced plateau. We demonstrate that the modified nonlinear spin wave (NLSW) approach indeed reproduces the magnetic excitation spectrum of Ba_3_CoSb_2_O_9_ within the 1/3 plateau^30–39^. The excellent agreement between theory and experiment demonstrates the semiclassical nature of magnons within the 1/3 plateau phase, despite the quantum fluctuation-induced nature of the ground state ordering. The resulting model parameters confirm that the anomalous zero-field dynamics reported in two independent experiments^37,39^ must be intrinsic and non-classical.

## Results

### Overview

**Fig. 1:**
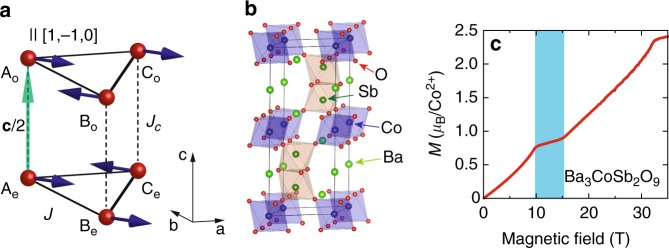
Stacked triangular lattice and the UUD state. **a** Spin structure in the quasi-2D lattice. **b** Crystal structure of Ba_3_CoSb_2_O_9_. **c** Magnetization curve for ${\mathbf{H}}||ab$ at *T* = 0.6 K highlighting the 1/3 plateau (the finite slope is due to Van Vleck paramagnetism^33^)

In this article, we present a comprehensive study of magnon excitations in the 1/3 magnetization plateau phase of a quasi-two-dimensional (quasi-2D) TLHAFM with easy-plane exchange anisotropy. Our study combines NLSW theory with in-field inelastic neutron scattering (INS) measurements of Ba_3_CoSb_2_O_9_. The Hamiltonian is

1 $$\begin{array}{*{20}{l}} {\it{{\cal H}}} \hfill & = \hfill & {J\mathop {\sum}\limits_{\langle {\mathbf{rr}}\prime \rangle } \left( {S_{\mathbf{r}}^xS_{{\mathbf{r}}\prime }^x + S_{\mathbf{r}}^yS_{{\mathbf{r}}\prime }^y + \Delta S_{\mathbf{r}}^zS_{{\mathbf{r}}\prime }^z} \right)} \hfill \\ {} \hfill & {} & \hskip-9pt{ + J_c\mathop {\sum}\limits_{\mathbf{r}} \left( {S_{\mathbf{r}}^xS_{{\mathbf{r}} + \frac{{\mathbf{c}}}{2}}^x + S_{\mathbf{r}}^yS_{{\mathbf{r}} + \frac{{\mathbf{c}}}{2}}^y + {\mathrm{\Delta }}S_{\mathbf{r}}^zS_{{\mathbf{r}} + \frac{{\mathbf{c}}}{2}}^z} \right) - h_{{\mathrm{red}}}\mathop {\sum}\limits_{\mathbf{r}} S_{\mathbf{r}}^x,} \hfill \end{array}$$

where 〈**rr**′〉 runs over in-plane nearest-neighbor (NN) sites of the stacked triangular lattice and $\frac{{\mathbf{c}}}{2}$ corresponds to the interlayer spacing (Fig. 1a). *J* (*J*_*c*_) is the antiferromagnetic intralayer (interlayer) NN exchange and 0 ≤ Δ < 1. The magnetic field is in the in-plane (*x*) direction (we use a spin-space coordinate frame where *x* and *y* are in the *ab* plane and *z* is parallel to *c*). *h*_red_ = *g*_⊥_*μ*_B_*H* is the reduced field and *g*_⊥_ is the in-plane *g*-tensor component.

This model describes Ba_3_CoSb_2_O_9_ (Fig. 1b), which comprises triangular layers of effective spin 1/2 moments arising from the ${\it{{\cal J}}} = 1/2$ Kramers doublet of Co^2+^ in a perfect octahedral ligand field. Excited multiplets are separated by a gap of 200–300 K due to spin–orbit coupling, which is much larger than the Néel temperature *T*_N_ = 3.8 K. Below *T* = *T*_N_, the material develops conventional 120° ordering with wavevector **Q** = (1/3, 1/3, 1)^30^. Experiments confirmed a 1/3 magnetization plateau for ${\mathbf{H}}||ab$ (Fig. 1c)^31,33,35,36,38^, which is robust down to the lowest temperatures. We compute the dynamical spin structure factor using NLSW theory in the 1/3 plateau phase. We also provide neutron diffraction evidence of the UUD state within the 1/3 plateau of Ba_3_CoSb_2_O_9_, along with maps of the excitation spectrum obtained from INS.

### Quantum-mechanical stabilization of the plateau in quasi-2D TLHAFMs

While experimental observations show that deviations from the ideal 2D TLHAFM are small in Ba_3_CoSb_2_O_9_^33,37,39^, a simple variational analysis shows that any *J*_*c*_ > 0 is enough to destabilize the UUD state classically. Thus, a naive spin wave treatment leads to instability for *J*_*c*_ > 0. However, the gapped nature of the spectrum^5^ implies that this phase must have a finite range of stability in quasi-2D materials. This situation must be quite generic among fluctuation-induced plateaus, as they normally require special conditions to be a classical ground state^9,10,43^.

**Fig. 2:**
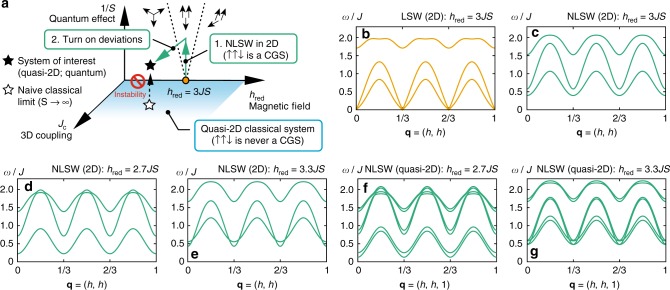
Scheme of NLSW theory for the 1/3 magnetization plateau in the quasi-2D TLHAFM. **a** Illustration of the procedure used to compute the spectrum. The filled and open star symbols represent the target quasi-2D quantum system and its naive classical limit, respectively. The spin structures favored by quantum fluctuation are shown on the *J*_*c*_ = 0 plane. **b** LSW spectrum along the high-symmetry direction of the Brillouin zone evaluated for *S* = 1/2, *J*_*c*_ = 0, Δ = 0.85 and *h*_red_ = 3*JS*, where the UUD state is a classical ground state (CGS). **c**–**e** NLSW spectra for *J*_*c*_ = 0 and Δ = 0.85 with (**c**) *h*_red_ = 3*JS*, (**d**) *h*_red_ = 2.7*JS*, and (**e**) *h*_red_ = 3.3*JS*. **f**, **g** NLSW spectra for *J*_*c*_/*J* = 0.09 and Δ = 0.85 with (**f**) *h*_red_ = 2.7*JS* and (**g**) *h*_red_ = 3.3*JS*

To put this into a proper semiclassical framework, we apply Alicea et al.'s trick originally applied to a distorted triangular lattice^40^. Basically, we make a detour in the parameter space with the additional 1/*S*-axis quantifying the quantum effect (Fig. 2). Namely, instead of expanding the Hamiltonian around *S* → ∞ for the actual model parameters, we start from the special point, *J*_*c*_ = 0, *h*_red_ = 3*JS*, and a given value of 0 ≤ Δ ≤ 1, where the UUD state is included in the classical ground state manifold. Assuming the spin structure in Fig. 1a, we define

2 $$S_{\mathbf{r}}^x = \tilde S_{\mathbf{r}}^z,S_{\mathbf{r}}^y = \tilde S_{\mathbf{r}}^y,S_{\mathbf{r}}^z = - \tilde S_{\mathbf{r}}^x,$$

for **r** ∈ A_e_, B_e_, A_o_, and C_o_ and

3 $$S_{\mathbf{r}}^x = - \tilde S_{\mathbf{r}}^z,S_{\mathbf{r}}^y = \tilde S_{\mathbf{r}}^y,S_{\mathbf{r}}^z = \tilde S_{\mathbf{r}}^x,$$

for **r** ∈ C_e_, B_o_. Introducing the Holstein–Primakoff bosons, $a_{\mu ,{\mathbf{r}}}^{\left( \dagger \right)}$, with 1 ≤ *μ* ≤ 6 being the sublattice index for A_e_, B_e_, C_e_, A_o_, B_o_, and C_o_ in this order, we have

4 $$\begin{array}{*{20}{l}} {\tilde S_{\mathbf{r}}^z} \hfill & = \hfill & {S - a_{\mu ,{\mathbf{r}}}^\dagger a_{\mu ,{\mathbf{r}}},} \hfill \\ {\tilde S_{\mathbf{r}}^ + } \hfill & = \hfill & {\tilde S_{\mathbf{r}}^x + {\rm i}\tilde S_{\mathbf{r}}^y \approx \sqrt {2S} \left( {1 - \frac{{a_{\mu ,{\mathbf{r}}}^\dagger a_{\mu ,{\mathbf{r}}}}}{{4S}}} \right)a_{\mu ,{\mathbf{r}}},} \hfill \end{array}$$

and $\tilde S_{\mathbf{r}}^ - = \left( {\tilde S_{\mathbf{r}}^ + } \right)^\dagger$ for **r** ∈ *μ*, truncating higher order terms irrelevant for the quartic interaction. We evaluate magnon self-energies arising from decoupling of the quartic term.

**Fig. 3:**
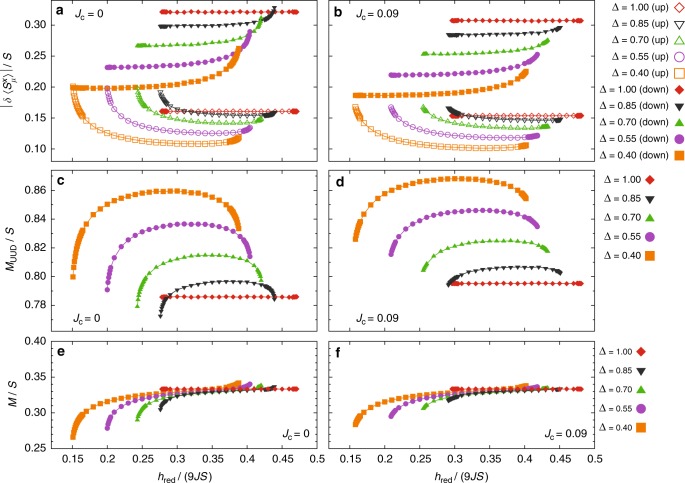
Calculated ordered moment within the 1/3 plateau for spin 1/2. Top row: magnitude of the reduction, $|\delta \langle S_\mu ^x\rangle |$, of the sublattice ordered moments (normalized by *S*) with the designated moment directions (up or down) for (**a**) *J*_*c*_ = 0 and (**b**) *J*_*c*_ = 0.09*J*; for sublattices with up spins, we average $\delta \langle S_\mu ^x\rangle$ over the corresponding two sublattices, discarding the small variance that appears for *J*_*c*_ > 0. Middle row: normalized staggered magnetization *M*_UUD_/*S* [Eq. (5)] for (**c**) *J*_*c*_ = 0 and (**d**) *J*_*c*_ = 0.09*J*. Bottom row: normalized (uniform) magnetization *M*/*S* for (**e**) *J*_*c*_ = 0 and (**f**) *J*_*c*_ = 0.09*J*. The results correspond to the local stability range of the plateau (with the precision 0.001 × 9*JS* for *h*_red_)

As shown in Fig. 2b, the linear spin wave (LSW) spectrum for *J*_*c*_ = 0 and *h*_red_ = 3*JS* features two *q*-linear gapless branches at **q** = 0, both of which are gapped out by the magnon–magnon interaction (Fig. 2c). Small deviations from *J*_*c*_ = 0 and *h*_red_ = 3*JS* do not affect the local stability of the UUD state because the gap must close continuously. Thus, we can investigate the excitation spectrum of quasi-2D systems for fields near *h*_red_ = 3*JS*. Figure 2d, e show the spectra for *h*_red_ shifted by ±10% from *h*_red_ = 3*JS*, where we still keep *J*_*c*_ = 0. For *h*_red_ < 3*JS*, a band-touching and subsequent hybridization appear between the middle and the top bands around **q** = (1/6,1/6) (Fig. 2d). For *h*_red_ > 3*JS*, a level-crossing between the middle and bottom bands appears at around **q** = 0 (Fig. 2e). A small *J*_*c*_ > 0 splits the three branches into six (Fig. 2f, g). Figure 3a, b show the reduction of the sublattice ordered moments for *S* = 1/2, *J*_*c*_ = 0, 0.09*J*, and selected values of Δ. We find $\left| {\delta \left\langle {S_\mu ^x} \right\rangle } \right|/S \lesssim 30\%$ throughout the local stability range of the plateau. Thus, our semiclassical approach is fully justified within the plateau phase. Figure 3c, d show the field dependence of the staggered magnetization,

5 $$M_{{\mathrm{UUD}}} = \frac{1}{6}\left( {\left\langle {S_{{\mathrm{A}}_{\mathrm{e}}}^x} \right\rangle \! + \! \left\langle {S_{{\mathrm{B}}_{\mathrm{e}}}^x} \right\rangle \! - \! \left\langle {S_{{\mathrm{C}}_{\mathrm{e}}}^x} \right\rangle \! + \! \left\langle {S_{{\mathrm{A}}_{\mathrm{o}}}^x} \right\rangle \! - \! \left\langle {S_{{\mathrm{B}}_{\mathrm{o}}}^x} \right\rangle \! + \! \left\langle {S_{{\mathrm{C}}_{\mathrm{o}}}^x} \right\rangle } \right),$$

which is almost field-independent; a slightly enhanced field-independence appears for small Δ. Similarly, while the magnetization is not conserved for Δ ≠ 1, it is nearly pinned at 1/3 for the most part of the plateau (Fig. 3e, f).

### UUD state in Ba_3_CoSb_2_O_9_

**Fig. 4:**
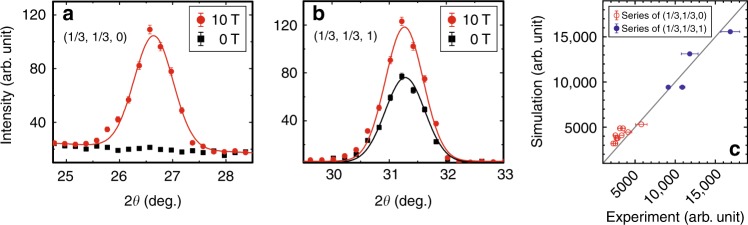
Neutron diffraction data for Ba_3_CoSb_2_O_9_ at 0 and 10 T. (**a**) **q** = (1/3, 1/3, 0) and (**b**) **q** = (1/3, 1/3, 1) measured at *T* = 1.5 K. **c** Comparison of the diffraction intensities between the experiment and the simulation at *T* = 1.5 K and *μ*_0_*H* = 10 T (the solid line is a guide to the eye). Error bars represent one standard deviation

Next we show experimental evidence for the UUD state in Ba_3_CoSb_2_O_9_ by neutron diffraction measurements within the plateau phase for field applied along the [1,–1,0] direction. We used the same single crystals reported previously^32,37^, grown by the traveling-solvent floating-zone technique and characterized by neutron diffraction, magnetic susceptibility, and heat capacity measurements. The space group is *P*6_3_/*mmc*, with the lattice constants *a* = *b* = 5.8562 Å and *c* = 14.4561 Å. The site-disorder between Co^2+^ and Sb^5+^ is negligible with the standard deviation of 1%, as reported elsewhere^37^. The magnetic and structural properties are consistent with previous reports and confirm high quality of the crystals^30–39^. These crystals were oriented in the (*h*, *h*, *l*) scattering plane. The magnetic Bragg peaks at (1/3, 1/3, 0) and (1/3, 1/3, 1) were measured at *T* = 1.5  K (Fig. 4a, b). The large intensity at both (1/3, 1/3, 0) and (1/3, 1/3, 1) confirms the UUD state at *μ*_0_*H* ≥ 9.8 T^32^ (Fig. 4c). The estimated ordered moment is 1.65(3) *μ*_B_ at 10 T and 1.80(9) *μ*_B_ at 10.9 T. They correspond to 85(2)% and 93(5)% of the full moment^33^, roughly coinciding with the predicted range (Fig. 3d). This diffraction pattern can be contrasted with that of the 120° state, characterized by a combination of the large intensity at (1/3, 1/3, 1) and lack of one at (1/3, 1/3, 0). Our diffraction result is fully consistent with previous nuclear magnetic resonance (NMR)^35^ and magnetization measurements^31,33^.

### Excitation spectrum

**Fig. 5:**
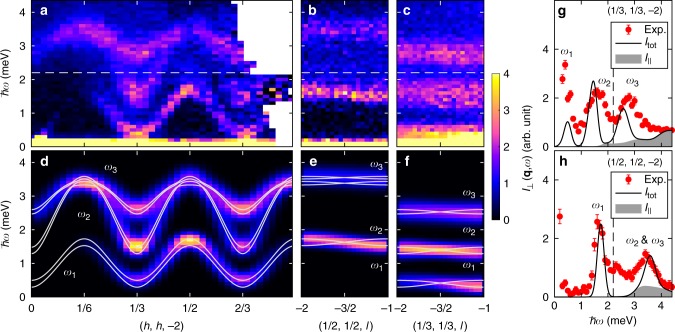
Excitation spectrum in the UUD phase of Ba_3_CoSb_2_O_9_. **a**–**c** Experimental scattering intensity at *μ*_0_*H* = 10.5 T and *T* = 0.5 K with momentum transfer (**a**) **q** = (*h*, *h*, −2), (**b**) **q** = (1/2, 1/2, *l*), and (**c**) **q** = (1/3, 1/3, *l*). **d**–**f** Calculated transverse part of the scattering intensity, *I*_⊥_(**q**, *ω*), obtained by NLSW theory along the same momentum cuts as in (**a**)–(**c**) for *J* = 1.74 meV, Δ = 0.85, *J*_*c*_/*J* = 0.09, and *g*_⊥_ = 3.95. The solid lines show the magnon poles. (**g**) and (**h**) Energy dependence of the calculated scattering intensity, *I*_tot_(**q**, *ω*) (solid line), compared with the experiment for (**g**) **q** = (1/3, 1/3, −2) and (**h**) **q** = (1/2, 1/2, −2) (error bars represent one standard deviation). The longitudinal contribution to the scattering intensity, $I_{||}\left( {{\mathbf{q}},\omega } \right)$, is plotted separately as a shaded area. The energy of the outgoing neutrons is *E*_f_ = 5 meV (3 meV) above (below) the dashed line in (**a**–**c**), (**g**), and (**h**)

We now turn to the dynamical properties in the UUD phase. Figure 5a–c show the INS intensity *I*(**q**, *ω*) ≡ *k*_i_/*k*_f_ (d^2^*σ*/dΩd*E*_f_) along high-symmetry directions. The applied magnetic field *μ*_0_*H* = 10.5 T is relatively close to the transition field *μ*_0_*H*_c1_ = 9.8 T^32^ bordering on the low-field coplanar ordered phase^35^, while the temperature *T* = 0.5 K is low enough compared to *T*_N_ ≈ 5 K^36^ for the UUD phase at this magnetic field. The in-plane dispersion shown in Fig. 5a comprises a seemingly gapless branch at **q** = (1/3, 1/3, −1) (Fig. 5c), and two gapped modes centered around 1.6 and 2.7 meV. Due to the interlayer coupling, each mode corresponds to two non-degenerate branches. As their splitting is below the instrumental resolution, we simply refer to them as *ω*_1_, *ω*_2_ and *ω*_3_, unless otherwise mentioned (Fig. 5). The dispersions along the *c*-direction are nearly flat, as shown in Fig. 5b, c for **q** = (1/2, 1/2, l) and **q** = (1/3, 1/3, *l*), respectively, reflecting the quasi-2D lattice^33,37^. Among the spin wave modes along **q** = (1/2, 1/2, *l*) and **q** = (1/3, 1/3, *l*) in Fig. 5b, c, *ω*_1_ for **q** = (1/2, 1/2, *l*) displays a relatively sharp spectral line. As discussed below, most of the broadening stems from the different intensities of the split modes due to finite *J*_*c*_.

**Fig. 6:**
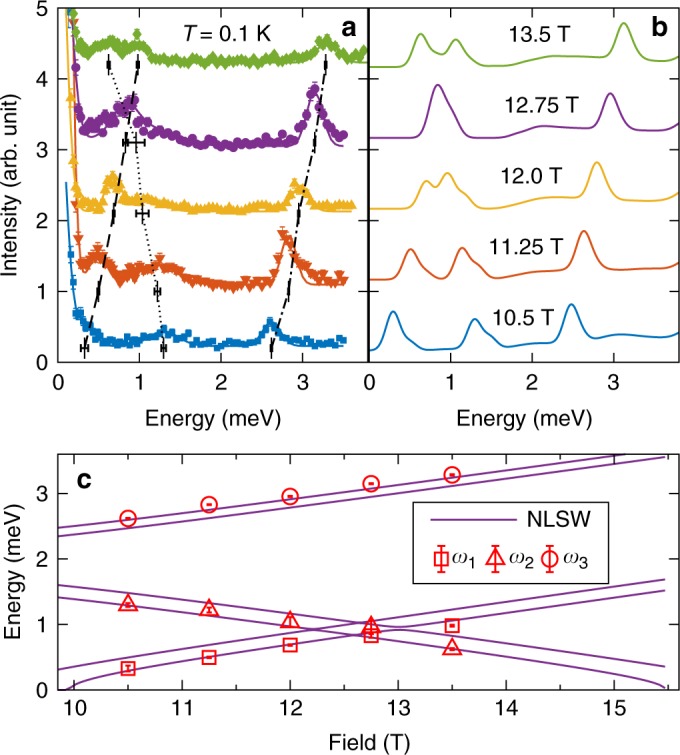
Field-dependence of *ω*_1_–*ω*_3_ at **q** = (1/3, 1/3, 1) in the UUD phase of Ba_3_CoSb_2_O_9_. **a** Constant-*q* scans for the selected values of the magnetic field at *T* = 0.1 K. The solid-lines show the fitting to Gaussian functions. The locations of the magnon peaks are indicated, where the solid (*ω*_1_), dashed (*ω*_2_), and dotted (*ω*_3_) lines are guides to the eye. **b** Simulation of the line-shape by NLSW theory for *J* = 1.74 meV, Δ = 0.85, *J*_*c*_/*J* = 0.09, and *g*_⊥_ = 3.95 convoluted with the assumed resolution 0.2 meV. **c** Comparison of the fitted magnon frequencies *ω*_1_–*ω*_3_ against the NLSW poles. Error bars represent one standard deviation

Comparing the experiment against the NLSW calculation, we find that the features of the in-plane spectrum in Fig. 5a are roughly captured by the theoretical calculation near the low-field onset of the plateau in Fig. 2f (*h*_red_ = 2.7*JS* ≈ 1.03*h*_red,c1_). This observation is in accord with the fact that the applied field (*μ*_0_*H* = 10.5 T) is close to *μ*_0_*H*_c1_ = 9.8 T^32^. To refine the quantitative comparison, we calculate the scattering intensity $I_{{\mathrm{tot}}}\left( {{\mathbf{q}},\omega } \right) \equiv \left( {\gamma r_0/2} \right)^2\left| {F\left( {\mathbf{q}} \right)} \right|^2\mathop {\sum}\nolimits_\alpha \left( {1 - \hat q^\alpha \hat q^\alpha } \right)g_\alpha ^2{\it{{\cal S}}}^{\alpha \alpha }\left( {{\mathbf{q}},\omega } \right)$ where *F*(**q**) denotes the magnetic form factor of Co^2+^ corrected with the orbital contribution, (*γr*_0_/2)^2^ is a constant, $\hat q^\alpha = q^\alpha /\left| {\mathbf{q}} \right|$ are the diagonal components of the dynamical structure factor evaluated at 10.5 T; off-diagonal components are zero due to symmetry. Defining the UUD order as shown in Fig. 1a, transverse spin fluctuations related to single-magnon excitations appear in ${\it{{\cal S}}}^{yy}$ and ${\it{{\cal S}}}^{zz}$, while longitudinal spin fluctuations corresponding to the two-magnon continuum appear in the inelastic part of ${\it{{\cal S}}}^{xx}$, denoted as ${\it{{\cal S}}}_{||}$. Accordingly, *I*_tot_(**q**, *ω*) can be separated into transverse *I*_⊥_ and longitudinal $I_{||}$ contributions. To compare with our experiments, the theoretical intensity is convoluted with momentum binning effects (only for *I*_⊥_) and empirical instrumental energy resolution. Figure 5d–f show the calculated *I*_⊥_(**q**, *ω*), along the same high-symmetry paths as the experimental results in Fig. 5a–c, for *J* = 1.74 meV, Δ = 0.85, *J*_*c*_/*J* = 0.09, and *g*_⊥_ = 3.95. The agreement between theory and experiment is excellent. When deriving these estimates, *J* is controlled by the saturation field *μ*_0_*H*_sat_ = 32.8 T for ${\mathbf{H}}||\hat c$^33^. To obtain the best fit, we also analyzed the field dependence of *ω*_1_, *ω*_2_, and *ω*_3_ (Fig. 6).

Remarkably, the calculation in Fig. 5d–f reproduces the dispersions almost quantitatively. It predicts a gapped *ω*_1_ mode, although the gap is below experimental resolution. The smallness of the gap is simply due to proximity to *H*_c1_. For each *ω*_*i*_, the band splitting due to *J*_*c*_ yields pairs of poles $\omega _i^ \pm$ dispersing with a phase difference of *π* in the out-of-triangular-plane direction (Fig. 5e, f). For each pair, however, one pole has a vanishing intensity for **q** = (1/2, 1/2, *l*). Consequently, *ω*_1_ along this direction is free from any extrinsic broadening caused by overlapping branches (Fig. 5e), yielding a relatively sharp spectral line (Fig. 5h). The corresponding bandwidth ≈ 0.2 meV (Fig. 5b) provides a correct estimate for *J*_*c*_. By contrast, for **q** = (1/3, 1/3, *l*), all six $\omega _i^ \pm$ branches have non-zero intensity, which leads to broadened spectra and less obvious dispersion along *l* (Fig. 5c, f).

The field-dependence of *ω*_1_–*ω*_3_ at **q** = (1/3, 1/3, 1) is extracted from constant-*q* scans at *T* = 0.1 K for selected fields 10.5–13.5 T within the plateau (Fig. 6a). By fitting the field-dependence of the low-energy branches of *ω*_1,2_, which become gapless at a plateau edge, we obtain the quoted model parameters. The field dependence is reproduced fairly well (Fig. 6b, c), although the calculation slightly underestimates *ω*_3_. We find *ω*_1_ and *ω*_3_ (*ω*_2_) increase (decreases) almost linearly in *H*, while the *ω*_1_ and *ω*_2_ branches cross around 12.6 T. The softening of *ω*_1_ (*ω*_2_) at the lower (higher) transition field induces the Y-like (V-like) state, respectively^20,35^. The nonlinearity of the first excitation gap near these transitions (visible only in the calculation) is due to the anisotropy; there is no U(1) symmetry along the field direction for Δ ≠ 1.

## Discussion

Our work has mapped out the excitation spectrum in the 1/3 plateau—a manifestation of quantum order-by-disorder effect—in Ba_3_CoSb_2_O_9_. Despite the quantum-mechanical origin of the ground state ordering, we have unambiguously demonstrated the semiclassical nature of magnons in this phase. In fact, the calculated reduction of the sublattice magnetization, *δS*_*μ*_ = *S* − |〈**S**_**r**_〉| with **r** ∈ *μ*, is relatively small (Fig. 3b): $\delta S_{{\mathrm{A}}_{\mathrm{e}}} = \delta S_{{\mathrm{A}}_{\mathrm{o}}} = 0.083$, $\delta S_{{\mathrm{B}}_{\mathrm{e}}} = \delta S_{{\mathrm{C}}_{\mathrm{o}}} = 0.073$, and $\delta S_{{\mathrm{C}}_{\mathrm{e}}} = \delta S_{{\mathrm{B}}_{\mathrm{o}}} = 0.14$ at 10.5 T for the quoted model parameters. This is consistent with the very weak intensity of the two-magnon continuum (Fig. 5g, h). This semiclassical behavior is protected by the excitation gap induced by anharmonicity of the spin waves (magnon–magnon interaction). We note that a perfect collinear magnetic order does not break any continuous symmetry even for Δ = 1, i.e., there is no gapless Nambu–Goldstone mode. The collinearity also means that three-magnon processes are not allowed^44^. The gap is robust against perturbations, such as anisotropies, lattice deformations^40^, or biquadratic couplings for *S* > 1/2 (a ferroquadrupolar coupling can stabilize the plateau even classically^5^). Thus, we expect the semiclassical nature of the excitation spectrum to be common to other 2D and quasi-2D realizations of fluctuation-induced plateaus, such as the 1/3 plateau in the spin-5/2 material RbFe(MoO_4_)_2_^29^. Meanwhile, it will be interesting to examine the validity of the semiclassical approach in quasi-1D TLHAFMs, such as Cs_2_CuBr_4_^23^, where quantum fluctuations are expected to be stronger.

Finally, we discuss the implications of our results for the zero-field dynamical properties of the same material, where recent experiments revealed unexpected phenomena, such as broadening of the magnon peaks indescribable by conventional spin-wave theory, large intensity of the high-energy continuum^37^, and the extension thereof to anomalously high frequencies^37,39^. Specifically, it was reported that magnon spectral-line broadened throughout the entire Brillouin zone, significantly beyond instrumental resolution, and a high frequency (≳ 2 meV) excitation continuum with an almost comparable spectral weight as single-magnon modes^37^. All of these experimental observations indicate strong quantum effects. Given that the spin Hamiltonian has been reliably determined from our study of the plateau phase, it is interesting to reexamine if a semiclassical treatment of this Hamiltonian can account for the zero-field anomalies.

**Fig. 7:**
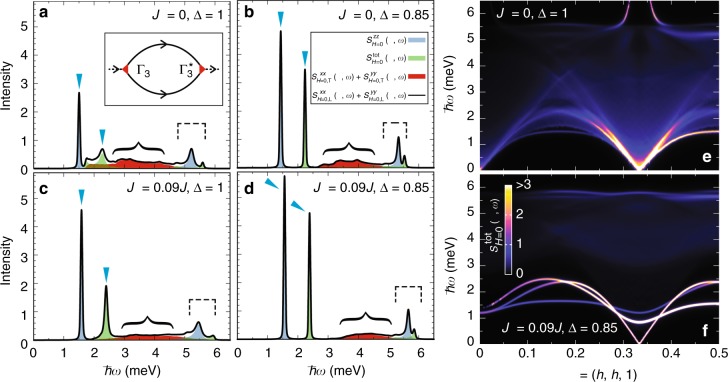
Calculated dynamical spin structure factor of the in-plane 120° state at *H* = 0. The calculations are made using NLSW theory for spin 1/2. **a**–**d** The results of the frequency dependence at **q** = (1/2, 1/2, 1) (M point) for (**a**) *J*_*c*_ = 0 and Δ = 1 (the ideal TLHAFM), (**b**) *J*_*c*_ = 0 and Δ = 0.85, (**c**) *J*_*c*_ = 0.09*J* and Δ = 1, and (**d**) *J*_*c*_ = 0.09*J* and Δ = 0.85. The results are convoluted with the energy resolution 0.015*J*. The total spin structure factor ${\it{{\cal S}}}_{H = 0}^{{\mathrm{tot}}}\left( {{\mathbf{q}},\omega } \right)$ (solid line) is divided into different components; ${\it{{\cal S}}}_{H = 0}^{zz}\left( {{\mathbf{q}},\omega } \right)$ and ${\it{{\cal S}}}_{H = 0,{\kern 1pt} {\mathrm{T}}}^{xx}\left( {{\mathbf{q}},\omega } \right) + {\it{{\cal S}}}_{H = 0,{\kern 1pt} {\mathrm{T}}}^{yy}\left( {{\mathbf{q}},\omega } \right)$ are single-magnon contributions (“T” denotes the transverse part), while the longitudinal (L) part ${\it{{\cal S}}}_{H = 0,{\kern 1pt} {\mathrm{L}}}^{xx}\left( {{\mathbf{q}},\omega } \right) + {\it{{\cal S}}}_{H = 0,{\kern 1pt} {\mathrm{L}}}^{yy}\left( {{\mathbf{q}},\omega } \right)$ corresponds to the two-magnon continuum; single magnon peaks (two-magnon continua) are indicated by arrows (curly brackets), whereas the dashed square brackets indicate anti-bonding single-magnon contributions, which are expected to be broadened by higher-order effect in 1/*S*^48^. The inset shows the lowest-order, *O*(*S*^0^), magnon self-energy incorporating the decay process of a single magnon into two magnons. **e** and **f** Intensity plots of ${\it{{\cal S}}}_{H = 0}^{{\mathrm{tot}}}\left( {{\mathbf{q}},\omega } \right)$ along the high-symmetry direction in the Brillouin zone for (**e**) *J*_*c*_ = 0 and Δ = 1 and (**f**) *J*_*c*_ = 0.09*J* and Δ = 0.85. *J* = 1.74 meV is assumed

A semiclassical treatment can only explain the line broadening in terms of magnon decay^44–48^. NLSW theory at *H* = 0 describes the spin fluctuations around the 120° ordered state by incorporating single-to-two magnon decay at the leading order *O*(*S*^0^). At this order, the two-magnon continuum is evaluated by convoluting LSW frequencies. The self-energies include Hartree–Fock decoupling terms, as well as the bubble Feynman diagrams comprising a pair of cubic vertices $\Gamma _3\sim O\left( {S^{1/2}} \right)$^45–48^, with the latter computed with the off-shell treatment. The most crucial one corresponds to the single-to-two magnon decay (see the inset of Fig. 7a),

6 $$\Sigma \left( {{\mathbf{q}},\omega } \right) = \frac{1}{{2N}}\mathop {\sum}\limits_{\mathbf{k}} \frac{{\left| {\Gamma _3\left( {{\mathbf{k}},{\mathbf{q}} - {\mathbf{k}};{\mathbf{q}}} \right)} \right|^2}}{{\omega - \omega _{\mathbf{k}}^{H = 0} - \omega _{{\mathbf{q}} - {\mathbf{k}}}^{H = 0} + {\rm i}0}},$$

where $\omega _{\mathbf{k}}^{H = 0}$ denotes the zero-field magnon dispersion. We show the zero-field dynamical structure factor, ${\it{{\cal S}}}_{H = 0}^{{\mathrm{tot}}}\left( {{\mathbf{q}},\omega } \right)$, at the M point for representative parameters in Fig. 7a–d. The NLSW result for the ideal TLHAFM (*J*_*c*_ = 0 and Δ = 1) exhibits sizable broadening and a strong two-magnon continuum^45–48^ (see also Fig. 7e). However, a slight deviation from Δ = 1 renders the decay process ineffective because the kinematic condition, $\omega _{\mathbf{q}}^{H = 0} = \omega _{\mathbf{k}}^{H = 0} + \omega _{{\mathbf{q}} - {\mathbf{k}}}^{H = 0}$, can no longer be fulfilled in 2D for any decay vertex over the entire Brillouin zone if ${\mathrm{\Delta }} \lesssim 0.92$^45^. This situation can be inferred from the result for *J*_*c*_ = 0 and Δ = 0.85, where the two-magnon continuum is pushed to higher frequencies, detached from the single-magnon peaks. In fact, the sharp magnon lines are free from broadening. The suppression of decay results from gapping out one of the two Nambu–Goldstone modes upon lowering the Hamiltonian symmetry from SU(2) to U(1), which greatly reduces the phase space for magnon decay. The interlayer coupling renders the single-magnon peaks even sharper and the continuum even weaker (Fig. 7c, d).

To determine whether the anomalous zero-field spin dynamics can be explained by a conventional 1/*S* expansion, it is crucial to estimate Δ very accurately. Previous experiments reported Δ = 0.95 (low-field electron spin resonance experiments compared with LSW theory^33^) and Δ = 0.89 (zero-field INS experiments compared with NLSW theory^37^). However, the NLSW calculation reported a large renormalization of the magnon bandwidth (≈ 40% reduction relative to the LSW theory)^37^, suggesting that the previous estimates of Δ may be inaccurate. Particularly, given that Δ is extracted by fitting the induced gap $\propto \sqrt {1 - {\mathrm{\Delta }}}$, the LSW approximation underestimates 1 − Δ (deviation from the isotropic exchange) because it overestimates the proportionality constant^37^.

Figure 7d, f show ${\it{{\cal S}}}_{H = 0}^{{\mathrm{tot}}}\left( {{\mathbf{q}},\omega } \right)$ for *J*_*c*_/*J* = 0.09 and Δ = 0.85. We find that ${\it{{\cal S}}}_{H = 0}^{{\mathrm{tot}}}\left( {{\mathbf{q}},\omega } \right)$ remains essentially semiclassical, with sharp magnon lines and a weak continuum, which deviates significantly from the recent results of INS experiments^37,39^. We thus conclude that the Hamiltonian that reproduces the plateau dynamics fails to do so at *H* = 0 within the spin wave theory, even after taking magnon–magnon interactions into account at the 1/*S* level. We also mention that the breakdown of the kinematic condition for single-to-two magnon decay also implies the breakdown of the condition for magnon decay into an arbitrary number of magnons^44^. Thus, the semiclassical picture of weakly interacting magnons is likely inadequate to simultaneously explain the low-energy dispersions and the intrinsic incoherent features (such as the high-intensity continuum and the line-broadening) observed in Ba_3_CoSb_2_O_9_ at *H* = 0.

One may wonder if extrinsic effects can explain these experimental observations. It is possible for exchange disorder to produce continuous excitations as in the effective spin-1/2 triangular antiferromagnet YbMgGaO_4_^49^. However, our single crystals are the same high-quality samples reported previously^32,37^, essentially free from Co^2+^–Sb^5+^ site-disorder. Indeed, our crystals show only one sharp peak at 3.6 K in the zero-field specific heat^32^ in contrast to the previous reports of multiple peaks^31^, which may indicate multi-domain structure. Another possible extrinsic effect is the magnon–phonon coupling, that has been invoked to explain the measured spectrum of the spin-3/2 TLHAFM CuCrO_2_^50^. However, if that effect were present at zero field, it should also be present in the UUD state. The fact that Eq. (1) reproduces the measured excitation spectrum of the UUD state suggests that the magnon–phonon coupling is negligibly small (a similar line of reasoning can also be applied to the effect of disorder). Indeed, we have also measured the phonon spectrum of Ba_3_CoSb_2_O_9_ in zero field by INS and found no strong signal of magnon–phonon coupling. Our results then suggest that the dynamics of the spin-1/2 TLHAFM is dominated by intrinsic quantum mechanical effects that escape a semiclassical spin-wave description. This situation is analogous to the (*π*, 0) wave-vector anomaly observed in various spin-1/2 square-lattice Heisenberg antiferromagnets^51–55^, but now extending to the entire Brillouin zone in the triangular lattice. Given recent theoretical success on the square-lattice^56^, our results motivate new non-perturbative studies of the spin-1/2 TLHAFM.

## Methods

### Neutron scattering measurements

The neutron diffraction data under magnetic fields applied in the [1,–1,0] direction were obtained by using CG-4C cold triple-axis spectrometer with the neutron energy fixed at 5.0 meV at High Flux Isotope Reactor (HFIR), Oak Ridge National Laboratory (ORNL). The nuclear structure of the crystal was determined at the HB-3A four-circle neutron diffractometer at HFIR, ORNL and then was used to fit the nuclear reflections measured at the CG-4C to confirm that the data reduction is valid. Only the scale factor was refined for fitting the nuclear reflections collected at CG-4C and was also used to scale the moment size for the magnetic structure refinement. 14 magnetic Bragg peaks collected at CG-4C at 10 T were used for the magnetic structure refinement. The UUD spin configuration with the spins along the field direction was found to best fit the data. The nuclear and magnetic structure refinements were carried out using FullProf Suite^57^.

Our inelastic neutron scattering experiments were carried out with the multi axis crystal spectrometer (MACS)^58^ at NIST Center for Neutron Research (NCNR), NIST, and the cold neutron triple-axis spectrometer (V2-FLEXX)^59^ at Helmholtz-Zentrum Berlin (HZB). The final energies were fixed at 3 and 5 meV on the MACS and 3.0 meV on V2-FLEXX.

### Constraint on *J* due to the saturation field

An exact expression for the saturation field for ${\mathbf{H}}||\hat c$, *H*_sat_, can be obtained from the level crossing condition between the fully polarized state and the ground state in the single-spin–flip sector. From the corresponding expression, we obtain:

7 $$J = \frac{{g_{||}\mu _{\rm B}H_{{\mathrm{sat}}}S^{ - 1}}}{{3 + 6{\mathrm{\Delta }} + 2\left( {1 + {\mathrm{\Delta }}} \right)\left( {J_c/J} \right)}},$$

where $$g_{||} = 3.87$$ and *μ*_0_*H*_sat_ = 32.8 T^33^.

### Variational analysis on classical instability of the 1/3 plateau in quasi-2D TLHAFMs

**Fig. 8:**
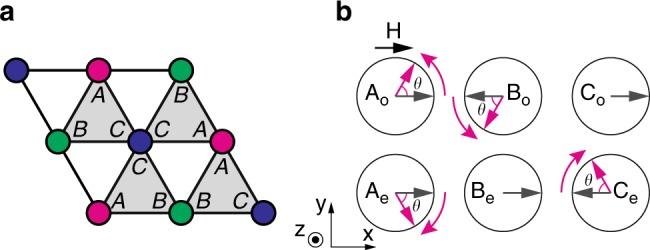
Classical instability of the UUD state in the quasi-2D lattice. **a** Three-sublattice structure for a single layer and a decomposition of the intralayer bonds into corner-sharing triangles. **b** Deformation of the UUD state (see Fig. 1a) parameterized by *θ* shown in the projection in the *ab* (or *xy*) plane; the spins in sublattices B_e_ and C_o_ are unchanged

We show that the UUD state is not the classical ground state in the presence of the antiferromagnetic interlayer exchange *J*_*c*_ > 0. To verify that the classical ground space for *J*_*c*_ = 0 acquires accidental degeneracy in the in-plane magnetic field, we rewrite Eq. (1) as

8 $$\begin{array}{*{20}{l}} {\it{{\cal H}}} \hfill & = \hfill & {\frac{J}{2}\mathop {\sum}\limits_{\mathrm{\Delta }} \left( {{\mathbf{S}}_{{\mathrm{\Delta }},{\mathrm{A}}} + {\mathbf{S}}_{{\mathrm{\Delta }},{\mathrm{B}}} + {\mathbf{S}}_{{\mathrm{\Delta }},{\mathrm{C}}} - \frac{{h_{{\mathrm{red}}}}}{{3J}}{\hat{\mathbf x}}} \right)^2} \hfill \\ {} \hfill & {} \hfill & { - \left( {1 - {\mathrm{\Delta }}} \right)J\mathop {\sum}\limits_{\left\langle {{\mathbf{rr}}\prime } \right\rangle } S_{\mathbf{r}}^zS_{{\mathbf{r}}\prime }^z + J_c\mathop {\sum}\limits_{\mathbf{r}} \left( {S_{\mathbf{r}}^xS_{{\mathbf{r}} + \frac{{{\hat{\mathbf c}}}}{2}}^x + S_{\mathbf{r}}^yS_{{\mathbf{r}} + \frac{{{\hat{\mathbf c}}}}{2}}^y + {\mathrm{\Delta }}S_{\mathbf{r}}^zS_{{\mathbf{r}} + \frac{{{\hat{\mathbf c}}}}{2}}^z} \right) + {\mathrm{const}}.,} \hfill \end{array}$$

where the summation of $\mathop {\sum}\nolimits_{\mathrm{\Delta }}$ is taken over the corner-sharing triangles in each layer, with **r** = (Δ, *μ*) (*μ* = A, B, C) denoting the sublattice sites in each triangle. This simply provides an alternative view of each triangular lattice layer (Fig. 8a). ${\hat{\mathbf x}}$ is the unit vector in the *x* or field direction. The easy-plane anisotropy forces every spin of the classical ground state to lie in the *ab* plane and the second term in Eq. (8) has no contribution at this level. Hence, for *J*_*c*_ = 0, any three-sublattice spin configuration satisfying $S_{\mathbf{r}}^z = 0$ and

9 $${\mathbf{S}}_{{\mathrm{\Delta }},{\mathrm{A}}} + {\mathbf{S}}_{{\mathrm{\Delta }},{\mathrm{B}}} + {\mathbf{S}}_{{\mathrm{\Delta }},{\mathrm{C}}} = \frac{{h_{{\mathrm{red}}}}}{{3J}}{\hat{\mathbf x}},\forall {\mathrm{\Delta }},$$

is a classical ground state, where we momentarily regard **S**_Δ,*μ*_ as three-component classical spins of length *S*. Since there are only two conditions corresponding to the *x* and *y* components of Eq. (9), whereas three angular variables are needed to specify the three-sublattice state in the *ab* plane, the classical ground state manifold for *J*_*c*_ = 0 retains an accidental degeneracy, similar to the well-known case of the Heisenberg model (Δ = 1)^43^. The UUD state is the classical ground state only for *h*_red_ = 3*JS*.

The classical instability of the UUD state for *J*_*c*_ > 0 can be demonstrated by a variational analysis. The UUD state in the 3D lattice enforces frustration for one- third of the antiferromagnetic interlayer bonds, inducing large variance of the interlayer interaction. As shown in Fig. 1a, only two of the three spin pairs along the **c**-axis per magnetic unit cell can be antiferromagnetically aligned, as favored by *J*_*c*_, while the last one has to be aligned ferromagnetically. To seek for a better classical solution, we consider a deformation of the spin configuration parameterized by 0 ≤ *θ* ≤ *π* at *h*_red_ = 3*JS*, such that the spin structure becomes noncollinear within the *ab* plane (Fig. 8b). Because the magnetization in each layer is fixed at *S*/3 per spin, the sum of the energies associated with the intralayer interaction and the Zeeman coupling is unchanged under this deformation. In the meantime, the energy per magnetic unit cell of the interlayer coupling is varied as

10 $$E_c(\theta ) = 2J_cS^2\left( {{\mathrm{cos}}\,2\theta - 2{\mathrm{cos}}\,\theta } \right).$$

We find that *E*_*c*_(*θ*) is minimized at *θ* = *π*/3 for *J*_*c*_ > 0, corresponding to a saddle point. This is a rather good approximation of the actual classical ground state for small *J*_*c*_ > 0, as can be demonstrated by direct minimization of the classical energy obtained from Eq. (1). The crucial observation is that the classical ground state differs from the *θ* = 0 UUD state.

### NLSW calculation for the UUD state

We summarize the derivation of the spin wave spectrum in the quasi-2D TLHAFM with easy-plane anisotropy [see Eq. (1)]. As discussed in the main text, we first work on the 2D limit *J*_*c*_ = 0 exactly at *h*_red_ = 3*JS*, and a given value of 0 ≤ Δ ≤ 1, which are the conditions for the UUD state to be the classical ground state. Defining the UUD state as shown in Fig. 1a, we introduce the Holstein–Primakoff bosons, $a_{\mu ,{\mathbf{r}}}^{(\dagger )}$ as in Eqs (2)–(4). After performing a Fourier transformation, $a_{\mu ,{\mathbf{k}}} = \left( {1/N_{{\mathrm{mag}}}} \right)^{1/2}\mathop {\sum}\nolimits_{{\mathbf{r}}\, \in \,\mu } {\rm e}^{ - {\rm i}{\mathbf{k}} \cdot {\mathbf{r}}}a_{\mu ,{\mathbf{r}}}$, where *N*_mag_ = *N*/6 is the number of magnetic unit cells (six spins for each) and *N* is the total number of spins, we obtain the quadratic Hamiltonian as the sum of even layers (sublattices A_e_–C_e_) and odd layers (sublattices A_o_–C_o_) contributions:

11 $${\it{{\cal H}}}_{{\mathrm{LSW}}}^0 = {\it{{\cal H}}}_{{\mathrm{LSW,even}}}^0 + {\it{{\cal H}}}_{{\mathrm{LSW,odd}}}^0,$$

where the constant term has been dropped. Here,

12 $${\it{{\cal H}}}_{{\mathrm{LSW,even}}}^0 = \frac{S}{2}\mathop {\sum}\limits_{{\mathbf{k}}\, \in \,{\mathrm{RBZ}}} {\left( {\left( {{\mathbf{a}}_{\mathbf{k}}^\dagger } \right)^T\left( {{\mathbf{a}}_{ - {\mathbf{k}}}} \right)^T} \right)} \left( {\begin{array}{*{20}{c}} {H_{11,{\mathbf{k}}}^0} & {H_{12,{\mathbf{k}}}^0} \\ {H_{21,{\mathbf{k}}}^0} & {H_{22,{\mathbf{k}}}^0} \end{array}} \right)\left( {\begin{array}{*{20}{c}} {{\mathbf{a}}_{\mathbf{k}}} \\ {{\mathbf{a}}_{ - {\mathbf{k}}}^\dagger } \end{array}} \right),$$

with $H_{11,{\mathbf{k}}}^0 = H_{22,{\mathbf{k}}}^0$, $H_{12,{\mathbf{k}}}^0 = H_{21,{\mathbf{k}}}^0$, where the summation over ***k*** is taken in the reduced Brillouin zone (RBZ) corresponding to the magnetic unit cell of the UUD state. From now on, we will denote this summation as $\mathop {\sum}\nolimits_{\mathbf{k}}$. We have introduced vector notation for the operators

13 $${\mathbf{a}}_{\mathbf{k}} = \left( {\begin{array}{*{20}{c}} {a_{{\mathrm{A}}_{\mathrm{e}},{\mathbf{k}}}} \\ {a_{{\mathrm{B}}_{\mathrm{e}},{\mathbf{k}}}} \\ {a_{{\mathrm{C}}_{\mathrm{e}},{\mathbf{k}}}} \end{array}} \right) \equiv \left( {\begin{array}{*{20}{c}} {a_{1,{\mathbf{k}}}} \\ {a_{2,{\mathbf{k}}}} \\ {a_{3,{\mathbf{k}}}} \end{array}} \right),{\mathbf{a}}_{ - {\mathbf{k}}}^\dagger = \left( {\begin{array}{*{20}{c}} {a_{{\mathrm{A}}_{\mathrm{e}}, - {\mathbf{k}}}^\dagger } \\ {a_{{\mathrm{B}}_{\mathrm{e}}, - {\mathbf{k}}}^\dagger } \\ {a_{{\mathrm{C}}_{\mathrm{e}}, - {\mathbf{k}}}^\dagger } \end{array}} \right) \equiv \left( {\begin{array}{*{20}{c}} {a_{1, - {\mathbf{k}}}^\dagger } \\ {a_{2, - {\mathbf{k}}}^\dagger } \\ {a_{3, - {\mathbf{k}}}^\dagger } \end{array}} \right),$$

and matrix notation for the quadratic coefficients

14 $$\begin{array}{l}\hskip-28pt H_{11,{\mathbf{k}}}^0 = \left( {\begin{array}{*{20}{c}} {S^{ - 1}h_{{\mathrm{red}}}} & {\frac{3}{2}J\left( {1 + \Delta } \right)\gamma _k} & {\frac{3}{2}J\left( {1 - \Delta } \right)\gamma _{ - k}} \\ {\frac{3}{2}J\left( {1 + {\mathrm{\Delta }}} \right)\gamma _{ - k}} & {S^{ - 1}h_{{\mathrm{red}}}} & {\frac{3}{2}J\left( {1 - \Delta } \right)\gamma _k} \\ {\frac{3}{2}J\left( {1 - {\mathrm{\Delta }}} \right)\gamma _k} & {\frac{3}{2}J\left( {1 - \Delta } \right)\gamma _{ - k}} & {6J - S^{ - 1}h_{{\mathrm{red}}}} \end{array}} \right),\\ H_{12,{\mathbf{k}}}^0 = \left( {\begin{array}{*{20}{c}} 0 & { - \frac{3}{2}J\left( {1 - \Delta } \right)\gamma _k} & { - \frac{3}{2}J\left( {1 + \Delta } \right)\gamma _{ - k}} \\ { - \frac{3}{2}J\left( {1 - \Delta } \right)\gamma _{ - k}} & 0 & { - \frac{3}{2}J\left( {1 + \Delta } \right)\gamma _k} \\ { - \frac{3}{2}J\left( {1 + \Delta } \right)\gamma _k} & { - \frac{3}{2}J\left( {1 + \Delta } \right)\gamma _{ - k}} & 0 \end{array}} \right),\end{array}$$

with $\gamma _{\mathbf{k}} = \frac{1}{3}\left( {{\rm e}^{{\rm i}{\mathbf{k}} \cdot {\mathbf{a}}} + {\rm e}^{{\rm i}{\mathbf{k}} \cdot {\mathbf{b}}} + {\rm e}^{ - {\rm i}{\mathbf{k}} \cdot \left( {{\mathbf{a}} + {\mathbf{b}}} \right)}} \right)$. Similarly, we have

15 $${\it{{\cal H}}}_{{\mathrm{LSW,odd}}}^0 = \frac{S}{2}\mathop {\sum}\limits_{\mathbf{k}} {\left( {\left( {{\bar{\mathbf a}}_{\mathbf{k}}^\dagger } \right)^{\mathrm{T}}\,\left( {{\bar{\mathbf a}}_{ - {\mathbf{k}}}} \right)^{\mathrm{T}}} \right)} \left( {\begin{array}{*{20}{c}} {\bar H_{11,{\mathbf{k}}}^0} & {\bar H_{12,{\mathbf{k}}}^0} \\ {\bar H_{21,{\mathbf{k}}}^0} & {\bar H_{22,{\mathbf{k}}}^0} \end{array}} \right)\left( {\begin{array}{*{20}{c}} {{\bar{\mathbf a}}_{\mathbf{k}}} \\ {{\bar{\mathbf a}}_{ - {\mathbf{k}}}^\dagger } \end{array}} \right),$$

with

16 $${\bar{\mathbf a}}_{\mathbf{k}} = \left( {\begin{array}{*{20}{c}} {a_{{\mathrm{A}}_{\mathrm{o}},{\mathbf{k}}}} \\ {a_{{\mathrm{B}}_{\mathrm{o}},{\mathbf{k}}}} \\ {a_{{\mathrm{C}}_{\mathrm{o}},{\mathbf{k}}}} \end{array}} \right) \equiv \left( {\begin{array}{*{20}{c}} {a_{4,{\mathbf{k}}}} \\ {a_{5,{\mathbf{k}}}} \\ {a_{6,{\mathbf{k}}}} \end{array}} \right),{\bar{\mathbf a}}_{ - {\mathbf{k}}}^\dagger = \left( {\begin{array}{*{20}{c}} {a_{{\mathrm{A}}_{\mathrm{o}}, - {\mathbf{k}}}^\dagger } \\ {a_{{\mathrm{B}}_{\mathrm{o}}, - {\mathbf{k}}}^\dagger } \\ {a_{{\mathrm{C}}_{\mathrm{o}}, - {\mathbf{k}}}^\dagger } \end{array}} \right) \equiv \left( {\begin{array}{*{20}{c}} {a_{4, - {\mathbf{k}}}^\dagger } \\ {a_{5, - {\mathbf{k}}}^\dagger } \\ {a_{6, - {\mathbf{k}}}^\dagger } \end{array}} \right),$$

and

17 $$\begin{array}{l}\bar H_{11,{\mathbf{k}}}^0 = \bar H_{22,{\mathbf{k}}}^0 = \left( {\begin{array}{*{20}{c}} 0 & 1 & 0 \\ 0 & 0 & 1 \\ 1 & 0 & 0 \end{array}} \right)H_{11,{\mathbf{k}}}^0\left( {\begin{array}{*{20}{c}} 0 & 0 & 1 \\ 1 & 0 & 0 \\ 0 & 1 & 0 \end{array}} \right),\\ \bar H_{12,{\mathbf{k}}}^0 = \bar H_{21,{\mathbf{k}}}^0 = \left( {\begin{array}{*{20}{c}} 0 & 1 & 0 \\ 0 & 0 & 1 \\ 1 & 0 & 0 \end{array}} \right)H_{12,{\mathbf{k}}}^0\left( {\begin{array}{*{20}{c}} 0 & 0 & 1 \\ 1 & 0 & 0 \\ 0 & 1 & 0 \end{array}} \right).\end{array}$$

The excitation spectrum of ${\it{{\cal H}}}_{{\mathrm{LSW}}}^0$ retains two **k**-linear modes at **k** = 0 (Fig. 2b). Below, we include nonlinear terms to gap out these excitations. At this stage, the nonlinear terms correspond to the mean-field (MF) decoupling of the intra-layer quartic terms. Once we obtain such a MF Hamiltonian with the gapped spectrum, the deviation from the fine-tuned magnetic field *h*_red_ = 3*JS* and interlayer coupling (as well as some other perturbation, if any) can be included. Here, the additional term contains both LSW and NLSW terms. To proceed, we first define the following mean-fields (MFs) symmetrized by using translational and rotational invariance:

18 $$\begin{array}{*{20}{l}} {\rho _\mu } \hfill & = \hfill & {\frac{1}{{N_{{\mathrm{mag}}}}}\mathop {\sum}\limits_{{\mathbf{r}}\, \in \,\mu } {\left\langle {a_{\mu ,{\mathbf{r}}}^\dagger a_{\mu ,{\mathbf{r}}}} \right\rangle _0} ,} \hfill \\ {\delta _\mu } \hfill & = \hfill & {\frac{1}{{N_{{\mathrm{mag}}}}}\mathop {\sum}\limits_{{\mathbf{r}}\, \in \,\mu } \left\langle {\left( {a_{\mu ,{\mathbf{r}}}} \right)^2} \right\rangle _0,} \hfill \\ {\xi _{\mu \nu }} \hfill & = \hfill & {\frac{1}{{3N_{{\mathrm{mag}}}}}\mathop {\sum}\limits_{{\mathbf{r}}\, \in \,\mu } \mathop {\sum}\limits_{\hat \eta _{\mu \nu }} \left\langle {a_{\mu ,{\mathbf{r}}}^\dagger a_{\nu ,{\mathbf{r}} + \hat \eta _{\mu \nu }}} \right\rangle _0,} \hfill \\ {\zeta _{\mu \nu }} \hfill & = \hfill & {\frac{1}{{3N_{{\mathrm{mag}}}}}\mathop {\sum}\limits_{{\mathbf{r}}\, \in \,\mu } \mathop {\sum}\limits_{\hat \eta _{\mu \nu }} \left\langle {a_{\mu ,{\mathbf{r}}}a_{\nu ,{\mathbf{r}} + \hat \eta _{\mu \nu }}} \right\rangle _0,} \hfill \end{array}$$

where $\hat \eta _{\mu \nu }$ represents the in-plane displacement vector connecting sites **r** ∈ *μ* to a nearest-neighbor site in sublattice *ν*. The mean values 〈...〉_0_ are evaluated with the ground state of ${\it{{\cal H}}}_{{\mathrm{LSW}}}^0$. The MFs for odd (even) layers are obtained from those for even (odd) layers as

19 $$\begin{array}{l}\rho _{{\mathrm{A}}_{\mathrm{o}}} = \rho _{{\mathrm{B}}_{\mathrm{e}}},\rho _{{\mathrm{B}}_{\mathrm{o}}} = \rho _{{\mathrm{C}}_{\mathrm{e}}},\rho _{{\mathrm{C}}_{\mathrm{o}}} = \rho _{{\mathrm{A}}_{\mathrm{e}}},\\ \delta _{{\mathrm{A}}_{\mathrm{o}}} = \delta _{{\mathrm{B}}_{\mathrm{e}}},\delta _{{\mathrm{B}}_{\mathrm{o}}} = \delta _{{\mathrm{C}}_{\mathrm{e}}},\delta _{{\mathrm{C}}_{\mathrm{o}}} = \delta _{{\mathrm{A}}_{\mathrm{e}}},\\ \xi _{{\mathrm{A}}_{\mathrm{o}}{\mathrm{B}}_{\mathrm{o}}} = \xi _{{\mathrm{B}}_{\mathrm{e}}{\mathrm{C}}_{\mathrm{e}}},\xi _{{\mathrm{B}}_{\mathrm{o}}{\mathrm{C}}_{\mathrm{o}}} = \xi _{{\mathrm{C}}_{\mathrm{e}}{\mathrm{A}}_{\mathrm{e}}},\xi _{{\mathrm{C}}_{\mathrm{o}}{\mathrm{A}}_{\mathrm{o}}} = \xi _{{\mathrm{A}}_{\mathrm{e}}{\mathrm{B}}_{\mathrm{e}}},\\ \zeta _{{\mathrm{A}}_{\mathrm{o}}{\mathrm{B}}_{\mathrm{o}}} = \zeta _{{\mathrm{B}}_{\mathrm{e}}{\mathrm{C}}_{\mathrm{e}}},\zeta _{{\mathrm{B}}_{\mathrm{o}}{\mathrm{C}}_{\mathrm{o}}} = \zeta _{{\mathrm{C}}_{\mathrm{e}}{\mathrm{A}}_{\mathrm{e}}},\zeta _{{\mathrm{C}}_{\mathrm{o}}{\mathrm{A}}_{\mathrm{o}}} = \zeta _{{\mathrm{A}}_{\mathrm{e}}{\mathrm{B}}_{\mathrm{e}}}.\end{array}$$

By collecting all the contributions mentioned above, we obtain the NLSW Hamiltonian

20 $${\it{{\cal H}}}_{{\mathrm{NLSW}}} =	 \frac{S}{2}\mathop {\sum}\limits_{\mathbf{k}} {\left( {\left( {{\mathbf{a}}_{\mathbf{k}}^\dagger } \right)^{\mathrm{T}}\,\left( {{\bar{\mathbf a}}_{\mathbf{k}}^\dagger } \right)^{\mathrm{T}}\,\left( {{\mathbf{a}}_{ - {\mathbf{k}}}} \right)^{\mathrm{T}}\,\left( {{\bar{\mathbf a}}_{ - {\mathbf{k}}}} \right)^{\mathrm{T}}} \right)} \\ 	\times\left( {\begin{array}{*{20}{c}} {H_{{\mathrm{ee}},{\mathbf{k}}}} & {H_{{\mathrm{eo}},{\mathbf{k}}}} & {H_{{\mathrm{ee}},{\mathbf{k}}}' } & {H_{{\mathrm{eo}},{\mathbf{k}}}' } \\ {\left( {H_{{\mathrm{eo}},{\mathbf{k}}}} \right)^\dagger } & {H_{{\mathrm{oo}},{\mathbf{k}}}} & {\left( {H_{{\mathrm{eo}}, - {\mathbf{k}}}' } \right)^{\mathrm{T}}} & {H_{{\mathrm{oo}},{\mathbf{k}}}' } \\ {\left( {H_{{\mathrm{ee}}, - {\mathbf{k}}}' } \right)^ \ast } & {\left( {H_{{\mathrm{eo}}, - {\mathbf{k}}}' } \right)^ \ast } & {\left( {H_{{\mathrm{ee}}, - {\mathbf{k}}}} \right)^ \ast } & {\left( {H_{{\mathrm{eo}}, - {\mathbf{k}}}} \right)^ \ast } \\ {\left( {H_{{\mathrm{eo}},{\mathbf{k}}}' } \right)^\dagger } & {\left( {H_{{\mathrm{oo}}, - {\mathbf{k}}}' } \right)^ \ast } & {\left( {H_{{\mathrm{eo}}, - {\mathbf{k}}}} \right)^{\mathrm{T}}} & {\left( {H_{{\mathrm{oo,}} - {\mathbf{k}}}} \right)^ \ast } \end{array}} \right)\left( {\begin{array}{*{20}{c}} {{\mathbf{a}}_{\mathbf{k}}} \\ {{\bar{\mathbf a}}_{\mathbf{k}}} \\ {{\mathbf{a}}_{ - {\mathbf{k}}}^\dagger } \\ {{\bar{\mathbf a}}_{ - {\mathbf{k}}}^\dagger } \end{array}} \right),$$

where

21 $$\begin{array}{l}\hskip-21pt H_{{\mathrm{e}}{{\mathrm{e}}},{\mathbf{k}}} = H_{11,{\mathbf{k}}}^0 + \left( {\begin{array}{*{20}{c}} { - 2J_c} & 0 & 0 \\ 0 & {2J_c} & 0 \\ 0 & 0 & {2J_c} \end{array}} \right) + S^{ - 1}\left( {\begin{array}{*{20}{c}} {\mu _{{\mathrm{MF}}}^{{\mathrm{A}}_{\mathrm{e}}} + 2J_c\rho _{{\mathrm{A}}_{\mathrm{o}}}} & {\left( {t_{{\mathrm{MF}}}^{{\mathrm{A}}_{\mathrm{e}}{\mathrm{B}}_{\mathrm{e}}}} \right)^ \ast \gamma _{\mathbf{k}}} & {t_{MF}^{{\mathrm{C}}_{\mathrm{e}}{\mathrm{A}}_{\mathrm{e}}}\gamma _{ - {\mathbf{k}}}} \\ {t_{{\mathrm{MF}}}^{{\mathrm{A}}_{\mathrm{e}}{\mathrm{B}}_{\mathrm{e}}}\gamma _{ - {\mathbf{k}}}} & {\mu ^{{\mathrm{B}}_{\mathrm{e}}}{MF}- 2J_c\rho _{{\mathrm{B}}_{\mathrm{o}}}} & {\left( {t_{{\mathrm{MF}}}^{{\mathrm{B}}_{\mathrm{e}}{\mathrm{C}}_{\mathrm{e}}}} \right)^ \ast \gamma _{\mathbf{k}}} \\ {\left( {t_{{\mathrm{MF}}}^{{\mathrm{C}}_{\mathrm{e}}{\mathrm{A}}_{\mathrm{e}}}} \right)^ \ast \gamma _{\mathbf{k}}} & {t_{{\mathrm{MF}}}^{{\mathrm{B}}_{\mathrm{e}}{\mathrm{C}}_{\mathrm{e}}}\gamma _{ - {\mathbf{k}}}} & {\mu _{{\mathrm{MF}}}^{{\mathrm{C}}_{\mathrm{e}}} - 2J_c\rho _{{\mathrm{C}}_{\mathrm{o}}}} \end{array}} \right),\\ \hskip-34pt H_{oo,{\mathbf{k}}} = \bar H_{11,{\mathbf{k}}}^0 + \left( {\begin{array}{*{20}{c}} { - 2J_c} & 0 & 0 \\ 0 & {2J_c} & 0 \\ 0 & 0 & {2J_c} \end{array}} \right) + S^{ - 1}\left( {\begin{array}{*{20}{c}} {\mu _{{\mathrm{MF}}}^{{\mathrm{A}}_{\mathrm{o}}} + 2J_c\rho _{{\mathrm{A}}_{\mathrm{e}}}} & {\left( {t_{{\mathrm{MF}}}^{{\mathrm{A}}_{\mathrm{o}}{\mathrm{B}}_{\mathrm{o}}}} \right)^ \ast \gamma _{\mathbf{k}}} & {t_{{\mathrm{MF}}}^{{\mathrm{C}}_{\mathrm{o}}{\mathrm{A}}_{\mathrm{o}}}\gamma _{ - {\mathbf{k}}}} \\ {t_{{\mathrm{MF}}}^{{\mathrm{A}}_{\mathrm{o}}{\mathrm{B}}_{\mathrm{o}}}\gamma _{ - {\mathbf{k}}}} & {\mu _{{\mathrm{MF}}}^{{\mathrm{B}}_{\mathrm{o}}} - 2J_c\rho _{{\mathrm{B}}_{\mathrm{e}}}} & {\left( {t_{{\mathrm{MF}}}^{{\mathrm{B}}_{\mathrm{o}}{\mathrm{C}}_{\mathrm{o}}}} \right)^ \ast \gamma _{\mathbf{k}}} \\ {\left( {t_{{\mathrm{MF}}}^{{\mathrm{C}}_{\mathrm{o}}{\mathrm{A}}_{\mathrm{o}}}} \right)^ \ast \gamma _{\mathbf{k}}} & {t_{{\mathrm{MF}}}^{{\mathrm{B}}_{\mathrm{o}}{\mathrm{C}}_{\mathrm{o}}}\gamma _{ - {\mathbf{k}}}} & {\mu _{{\mathrm{MF}}}^{{\mathrm{C}}_{\mathrm{o}}} - 2J_c\rho _{{\mathrm{C}}_{\mathrm{e}}}} \end{array}} \right),\\ \hskip-180pt H_{{\mathrm{ee,k}}}' = H_{12,{\mathbf{k}}}^0 + S^{ - 1}\left( {\begin{array}{*{20}{c}} {\Gamma _{{\mathrm{MF}}}^{{\mathrm{A}}_{\mathrm{e}}}} & {g_{{\mathrm{MF}}}^{{\mathrm{A}}_{\mathrm{e}}{\mathrm{B}}_{\mathrm{e}}}\gamma _{\mathbf{k}}} & {g_{{\mathrm{MF}}}^{{\mathrm{C}}_{\mathrm{e}}{\mathrm{A}}_{\mathrm{e}}}\gamma _{ - {\mathbf{k}}}} \\ {g_{{\mathrm{MF}}}^{{\mathrm{A}}_{\mathrm{e}}{\mathrm{B}}_{\mathrm{e}}}\gamma _{ - {\mathbf{k}}}} & {\Gamma _{{\mathrm{MF}}}^{{\mathrm{B}}_{\mathrm{e}}}} & {g_{{\mathrm{MF}}}^{{\mathrm{B}}_{\mathrm{e}}{\mathrm{C}}_{\mathrm{e}}}\gamma _{\mathbf{k}}} \\ {g_{{\mathrm{MF}}}^{{\mathrm{C}}_{\mathrm{e}}{\mathrm{A}}_{\mathrm{e}}}\gamma _{\mathbf{k}}} & {g_{{\mathrm{MF}}}^{{\mathrm{B}}_{\mathrm{e}}{\mathrm{C}}_{\mathrm{e}}}\gamma _{ - {\mathbf{k}}}} & {\Gamma _{{\mathrm{MF}}}^C} \end{array}} \right),\\ \hskip-177pt H_{{\mathrm{oo}},{\mathbf{k}}}' = \bar H_{12,{\mathbf{k}}}^0 + S^{ - 1}\left( {\begin{array}{*{20}{c}} {\Gamma _{{\mathrm{MF}}}^{{\mathrm{A}}_{\mathrm{o}}}} & {g_{{\mathrm{MF}}}^{{\mathrm{A}}_{\mathrm{o}}{\mathrm{B}}_{\mathrm{o}}}\gamma _{\mathbf{k}}} & {g_{{\mathrm{MF}}}^{{\mathrm{C}}_{\mathrm{o}}{\mathrm{A}}_{\mathrm{o}}}\gamma _{ - {\mathbf{k}}}} \\ {g_{{\mathrm{MF}}}^{{\mathrm{A}}_{\mathrm{o}}{\mathrm{B}}_{\mathrm{o}}}\gamma _{ - {\mathbf{k}}}} & {\Gamma _{{\mathrm{MF}}}^{{\mathrm{B}}_{\mathrm{o}}}} & {g_{{\mathrm{MF}}}^{{\mathrm{B}}_{\mathrm{o}}{\mathrm{C}}_{\mathrm{o}}}\gamma _{\mathbf{k}}} \\ {g_{{\mathrm{MF}}}^{{\mathrm{C}}_{\mathrm{o}}{\mathrm{A}}_{\mathrm{o}}}\gamma _{\mathbf{k}}} & {g_{{\mathrm{MF}}}^{{\mathrm{B}}_{\mathrm{o}}{\mathrm{C}}_{\mathrm{o}}}\gamma _{ - {\mathbf{k}}}} & {\Gamma _{{\mathrm{MF}}}^{{\mathrm{C}}_{\mathrm{o}}}} \end{array}} \right),\\ \hskip-1pt H_{{\mathrm{eo}},{\mathbf{k}}} = {\mathrm{cos}}k_3\left( {\begin{array}{*{20}{c}} {J_c\left( {1 + \Delta } \right)} & 0 & 0 \\ 0 & {J_c\left( {1 - {\mathrm{\Delta }}} \right)} & 0 \\ 0 & 0 & {J_c\left( {1 - {\mathrm{\Delta }}} \right)} \end{array}} \right) + S^{ - 1}{\mathrm{cos}}k_3\left( {\begin{array}{*{20}{c}} {\left( {t_{MF}^{{\mathrm{A}}_{\mathrm{e}}{\mathrm{A}}_{\mathrm{o}}}} \right)^ \ast } & 0 & 0 \\ 0 & {\left( {t_{MF}^{{\mathrm{B}}_{\mathrm{e}}{\mathrm{B}}_{\mathrm{o}}}} \right)^ \ast } & 0 \\ 0 & 0 & {\left( {t_{MF}^{{\mathrm{C}}_{\mathrm{e}}{\mathrm{C}}_{\mathrm{o}}}} \right)^ \ast } \end{array}} \right),\\ H_{{\mathrm{eo}},{\mathbf{k}}}' = {\mathrm{cos}}k_3\left( {\begin{array}{*{20}{c}} { - J_c\left( {1 - {\mathrm{\Delta }}} \right)} & 0 & 0 \\ 0 & { - J_c\left( {1 + {\mathrm{\Delta }}} \right)} & 0 \\ 0 & 0 & { - J_c\left( {1 + {\mathrm{\Delta }}} \right)} \end{array}} \right) + S^{ - 1}{\mathrm{cos}}k_3\left( {\begin{array}{*{20}{c}} {g_{{\mathrm{MF}}}^{{\mathrm{A}}_{\mathrm{e}}{\mathrm{A}}_{\mathrm{o}}}} & 0 & 0 \\ 0 & {g_{{\mathrm{MF}}}^{{\mathrm{B}}_{\mathrm{e}}{\mathrm{B}}_{\mathrm{o}}}} & 0 \\ 0 & 0 & {g_{{\mathrm{MF}}}^{{\mathrm{C}}_{\mathrm{e}}{\mathrm{C}}_{\mathrm{o}}}} \end{array}} \right).\hskip16pt\end{array}$$

Here the MF parameters are given as follows. First, those associated with the intralayer coupling are

22 $$\begin{array}{*{20}{l}} {\mu _{{\mathrm{MF}}}^{{\mathrm{A}}_{\mathrm{e}}}} \hfill & = \hfill & {3J\left[ {\rho _{{\mathrm{B}}_{\mathrm{e}}} - \rho _{{\mathrm{C}}_{\mathrm{e}}} - \frac{{1 + \Delta }}{2}\left( {{\mathrm{Re}}{\kern 1pt} \xi _{{\mathrm{A}}_{\mathrm{e}}{\mathrm{B}}_{\mathrm{e}}} - {\mathrm{Re}}{\kern 1pt} \zeta _{{\mathrm{C}}_{\mathrm{e}}{\mathrm{A}}_{\mathrm{e}}}} \right) - \frac{{1 - \Delta }}{2}\left( {{\mathrm{Re}}{\kern 1pt} \xi _{{\mathrm{C}}_{\mathrm{e}}{\mathrm{A}}_{\mathrm{e}}} - {\mathrm{Re}}{\kern 1pt} \zeta _{{\mathrm{A}}_{\mathrm{e}}{\mathrm{B}}_{\mathrm{e}}}} \right)} \right],} \hfill \\ {\mu _{{\mathrm{MF}}}^{{\mathrm{B}}_{\mathrm{e}}}} \hfill & = \hfill & {3J\left[ {\rho _{{\mathrm{A}}_{\mathrm{e}}} - \rho _{{\mathrm{C}}_{\mathrm{e}}} - \frac{{1 + \Delta }}{2}\left( {{\mathrm{Re}}{\kern 1pt} \xi _{{\mathrm{A}}_{\mathrm{e}}{\mathrm{B}}_{\mathrm{e}}} - {\mathrm{Re}}{\kern 1pt} \zeta _{{\mathrm{B}}_{\mathrm{e}}{\mathrm{C}}_{\mathrm{e}}}} \right) - \frac{{1 - \Delta }}{2}\left( {{\mathrm{Re}}{\kern 1pt} \xi _{{\mathrm{B}}_{\mathrm{e}}{\mathrm{C}}_{\mathrm{e}}} - {\mathrm{Re}}{\kern 1pt} \zeta _{{\mathrm{A}}_{\mathrm{e}}{\mathrm{B}}_{\mathrm{e}}}} \right)} \right],} \hfill \\ {\mu _{{\mathrm{MF}}}^{{\mathrm{C}}_{\mathrm{e}}}} \hfill & = \hfill & {3J\left[ { - \rho _{{\mathrm{A}}_{\mathrm{e}}} - \rho _{{\mathrm{B}}_{\mathrm{e}}} + \frac{{1 + \Delta }}{2}\left( {{\mathrm{Re}}{\kern 1pt} \zeta _{{\mathrm{B}}_{\mathrm{e}}{\mathrm{C}}_{\mathrm{e}}} + {\mathrm{Re}}{\kern 1pt} \zeta _{{\mathrm{C}}_{\mathrm{e}}{\mathrm{A}}_{\mathrm{e}}}} \right) - \frac{{1 - \Delta }}{2}\left( {{\mathrm{Re}}{\kern 1pt} \xi _{{\mathrm{B}}_{\mathrm{e}}{\mathrm{C}}_{\mathrm{e}}} + {\mathrm{Re}}{\kern 1pt} \xi _{{\mathrm{C}}_{\mathrm{e}}{\mathrm{A}}_{\mathrm{e}}}} \right)} \right],} \hfill \\ {t_{{\mathrm{MF}}}^{{\mathrm{A}}_{\mathrm{e}}{\mathrm{B}}_{\mathrm{e}}}} \hfill & = \hfill & {3J\left[ {\xi _{{\mathrm{A}}_{\mathrm{e}}{\mathrm{B}}_{\mathrm{e}}} - \frac{{1 + \Delta }}{4}\left( {\rho _{{\mathrm{A}}_{\mathrm{e}}} + \rho _{{\mathrm{B}}_{\mathrm{e}}}} \right) + \frac{{1 - \Delta }}{8}\left( {\delta _{{\mathrm{A}}_{\mathrm{e}}}^ \ast + \delta _{{\mathrm{B}}_{\mathrm{e}}}} \right)} \right],} \hfill \\ {t_{{\mathrm{MF}}}^{{\mathrm{B}}_{\mathrm{e}}{\mathrm{C}}_{\mathrm{e}}}} \hfill & = \hfill & {3J\left[ { - \xi _{{\mathrm{B}}_{\mathrm{e}}{\mathrm{C}}_{\mathrm{e}}} + \frac{{1 + \Delta }}{8}\left( {\delta _{{\mathrm{B}}_{\mathrm{e}}}^ \ast + \delta _{{\mathrm{C}}_{\mathrm{e}}}} \right) - \frac{{1 - \Delta }}{4}\left( {\rho _{{\mathrm{B}}_{\mathrm{e}}} + \rho _{{\mathrm{C}}_{\mathrm{e}}}} \right)} \right],} \hfill \\ {t_{{\mathrm{MF}}}^{{\mathrm{C}}_{\mathrm{e}}{\mathrm{A}}_{\mathrm{e}}}} \hfill & = \hfill & {3J\left[ { - \xi _{{\mathrm{C}}_{\mathrm{e}}{\mathrm{A}}_{\mathrm{e}}} + \frac{{1 + {\mathrm{\Delta }}}}{8}\left( {\delta _{{\mathrm{C}}_{\mathrm{e}}}^ \ast + \delta _{{\mathrm{A}}_{\mathrm{e}}}} \right) - \frac{{1 - {\mathrm{\Delta }}}}{4}\left( {\rho _{{\mathrm{C}}_{\mathrm{e}}} + \rho _{{\mathrm{A}}_{\mathrm{e}}}} \right)} \right],} \hfill \\ {\Gamma _{{\mathrm{MF}}}^{{\mathrm{A}}_{\mathrm{e}}}} \hfill & = \hfill & {\frac{{3J}}{2}\left[ {\frac{{1 + {\mathrm{\Delta }}}}{2}\left( {\xi _{{\mathrm{C}}_{\mathrm{e}}{\mathrm{A}}_{\mathrm{e}}} - \zeta _{{\mathrm{A}}_{\mathrm{e}}{\mathrm{B}}_{\mathrm{e}}}} \right) + \frac{{1 - {\mathrm{\Delta }}}}{2}\left( {\xi _{{\mathrm{A}}_{\mathrm{e}}{\mathrm{B}}_{\mathrm{e}}}^ \ast - \zeta _{{\mathrm{C}}_{\mathrm{e}}{\mathrm{A}}_{\mathrm{e}}}} \right)} \right],} \hfill \\ {\Gamma _{{\mathrm{MF}}}^{{\mathrm{B}}_{\mathrm{e}}}} \hfill & = \hfill & {\frac{{3J}}{2}\left[ {\frac{{1 + {\mathrm{\Delta }}}}{2}\left( {\xi _{{\mathrm{B}}_{\mathrm{e}}{\mathrm{C}}_{\mathrm{e}}}^ \ast - \zeta _{{\mathrm{A}}_{\mathrm{e}}{\mathrm{B}}_{\mathrm{e}}}} \right) + \frac{{1 - {\mathrm{\Delta }}}}{2}\left( {\xi _{{\mathrm{A}}_{\mathrm{e}}{\mathrm{B}}_{\mathrm{e}}} - \zeta _{{\mathrm{B}}_{\mathrm{e}}{\mathrm{C}}_{\mathrm{e}}}} \right)} \right]} \hfill \\ {\Gamma _{{\mathrm{MF}}}^{{\mathrm{C}}_{\mathrm{e}}}} \hfill & = \hfill & {\frac{{3J}}{2}\left[ {\frac{{1 + {\mathrm{\Delta }}}}{2}\left( {\xi _{{\mathrm{B}}_{\mathrm{e}}{\mathrm{C}}_{\mathrm{e}}}^ \ast - \xi _{{\mathrm{C}}_{\mathrm{e}}{\mathrm{A}}_{\mathrm{e}}}} \right) + \frac{{1 - {\mathrm{\Delta }}}}{2}\left( {\zeta _{{\mathrm{B}}_{\mathrm{e}}{\mathrm{C}}_{\mathrm{e}}} - \zeta _{{\mathrm{C}}_{\mathrm{e}}{\mathrm{A}}_{\mathrm{e}}}} \right)} \right],} \hfill \\ {g_{{\mathrm{MF}}}^{{\mathrm{A}}_{\mathrm{e}}{\mathrm{B}}_{\mathrm{e}}}} \hfill & = \hfill & {3J\left[ {\zeta _{{\mathrm{A}}_{\mathrm{e}}{\mathrm{B}}_{\mathrm{e}}} - \frac{{1 + {\mathrm{\Delta }}}}{8}\left( {\delta _{{\mathrm{A}}_{\mathrm{e}}} + \delta _{{\mathrm{B}}_{\mathrm{e}}}} \right) + \frac{{1 - {\mathrm{\Delta }}}}{4}\left( {\rho _{{\mathrm{A}}_{\mathrm{e}}} + \rho _{{\mathrm{B}}_{\mathrm{e}}}} \right)} \right],} \hfill \\ {g_{{\mathrm{MF}}}^{{\mathrm{B}}_{\mathrm{e}}{\mathrm{C}}_{\mathrm{e}}}} \hfill & = \hfill & {3J\left[ { - \zeta _{{\mathrm{B}}_{\mathrm{e}}{\mathrm{C}}_{\mathrm{e}}} + \frac{{1 + {\mathrm{\Delta }}}}{4}\left( {\rho _{\mathrm{B}} + \rho _{\mathrm{C}}} \right) - \frac{{1 - {\mathrm{\Delta }}}}{8}\left( {\delta _{\mathrm{B}} + \delta _{\mathrm{C}}} \right)} \right],} \hfill \\ {g_{{\mathrm{MF}}}^{{\mathrm{C}}_{\mathrm{e}}{\mathrm{A}}_{\mathrm{e}}}} \hfill & = \hfill & {3J\left[ { - \zeta _{{\mathrm{C}}_{\mathrm{e}}{\mathrm{A}}_{\mathrm{e}}} + \frac{{1 + {\mathrm{\Delta }}}}{4}\left( {\rho _{{\mathrm{C}}_{\mathrm{e}}} + \rho _{{\mathrm{A}}_{\mathrm{e}}}} \right) - \frac{{1 - {\mathrm{\Delta }}}}{8}\left( {\delta _{{\mathrm{C}}_{\mathrm{e}}} + \delta _{{\mathrm{A}}_{\mathrm{e}}}} \right)} \right],} \hfill \end{array}$$

for even layers and

23 $$\begin{array}{l}\hskip-4pt\mu _{{\mathrm{MF}}}^{{\mathrm{A}}_{\mathrm{o}}} = \mu _{{\mathrm{MF}}}^{{\mathrm{B}}_{\mathrm{e}}}, \mu _{{\mathrm{MF}}}^{{\mathrm{B}}_{\mathrm{o}}} = \mu _{{\mathrm{MF}}}^{{\mathrm{C}}_{\mathrm{e}}},\mu _{{\mathrm{MF}}}^{{\mathrm{C}}_{\mathrm{o}}} = \mu _{{\mathrm{MF}}}^{{\mathrm{A}}_{\mathrm{e}}},\\ \hskip9pt t_{MF}^{{\mathrm{A}}_{\mathrm{o}}{\mathrm{B}}_{\mathrm{o}}} = t_{MF}^{{\mathrm{B}}_{\mathrm{e}}{\mathrm{C}}_{\mathrm{e}}},t_{MF}^{{\mathrm{B}}_{\mathrm{o}}{\mathrm{C}}_{\mathrm{o}}} = t_{MF}^{{\mathrm{C}}_{\mathrm{e}}{\mathrm{A}}_{\mathrm{e}}}, t_{MF}^{{\mathrm{C}}_{\mathrm{o}}{\mathrm{A}}_{\mathrm{o}}} = t_{MF}^{{\mathrm{A}}_{\mathrm{e}}{\mathrm{B}}_{\mathrm{e}}},\\ \hskip-4pt \Gamma _{{\mathrm{MF}}}^{{\mathrm{A}}_{\mathrm{o}}} = \Gamma _{{\mathrm{MF}}}^{{\mathrm{B}}_{\mathrm{e}}},\Gamma _{{\mathrm{MF}}}^{{\mathrm{B}}_{\mathrm{o}}} = \Gamma _{{\mathrm{MF}}}^{{\mathrm{C}}_{\mathrm{e}}},\Gamma _{{\mathrm{MF}}}^{{\mathrm{C}}_{\mathrm{o}}} = \Gamma _{{\mathrm{MF}}}^{{\mathrm{A}}_{\mathrm{e}}},\\ \hskip15pt g_{{\mathrm{MF}}}^{{\mathrm{A}}_{\mathrm{o}}{\mathrm{B}}_{\mathrm{o}}} = g_{{\mathrm{MF}}}^{{\mathrm{B}}_{\mathrm{e}}{\mathrm{C}}_{\mathrm{e}}},g_{{\mathrm{MF}}}^{{\mathrm{B}}_{\mathrm{o}}{\mathrm{C}}_{\mathrm{o}}} = g_{{\mathrm{MF}}}^{{\mathrm{C}}_{\mathrm{e}}{\mathrm{A}}_{\mathrm{e}}},g_{{\mathrm{MF}}}^{{\mathrm{C}}_{\mathrm{o}}{\mathrm{A}}_{\mathrm{o}}} = g_{{\mathrm{MF}}}^{{\mathrm{A}}_{\mathrm{e}}{\mathrm{B}}_{\mathrm{e}}},\end{array}$$

for odd layers. Similarly, the new MF parameters associated with the interlayer coupling are

24 $$\begin{array}{l}t_{\mathrm{MF}}^{{\mathrm{A}}_{\mathrm{e}}{\mathrm{A}}_{\mathrm{o}}} = J_c\left[ { - \frac{{1 + \Delta }}{2}\left( {\rho _{{\mathrm{A}}_{\mathrm{e}}} + \rho _{{\mathrm{A}}_{\mathrm{o}}}} \right) + \frac{{1 - \Delta }}{4}\left( {\delta _{{\mathrm{A}}_{\mathrm{e}}}^ \ast + \delta _{{\mathrm{A}}_{\mathrm{o}}}} \right)} \right],\\ \hskip-11pt t_{\mathrm{MF}}^{{\mathrm{B}}_{\mathrm{e}}{\mathrm{B}}_{\mathrm{o}}} = J_c\left[ {\frac{{1 + \Delta }}{4}\left( {\delta _{{\mathrm{B}}_{\mathrm{e}}}^ \ast + \delta _{{\mathrm{B}}_{\mathrm{o}}}} \right) - \frac{{1 - \Delta }}{2}\left( {\rho _{{\mathrm{B}}_{\mathrm{e}}} + \rho _{{\mathrm{B}}_{\mathrm{o}}}} \right)} \right],\\ \hskip-10pt t_{\mathrm{MF}}^{{\mathrm{C}}_{\mathrm{e}}{\mathrm{C}}_{\mathrm{o}}} = J_c\left[ {\frac{{1 + \Delta }}{4}\left( {\delta _{{\mathrm{C}}_{\mathrm{e}}}^ \ast + \delta _{{\mathrm{C}}_{\mathrm{o}}}} \right) - \frac{{1 - \Delta }}{2}\left( {\rho _{{\mathrm{C}}_{\mathrm{e}}} + \rho _{{\mathrm{C}}_{\mathrm{o}}}} \right)} \right],\\ \hskip-1pt g_{\mathrm {MF}}^{{\mathrm{A}}_{\mathrm{e}}{\mathrm{A}}_{\mathrm{o}}} = J_c\left[ { - \frac{{1 + \Delta }}{4}\left( {\delta _{{\mathrm{A}}_{\mathrm{e}}} + \delta _{{\mathrm{A}}_{\mathrm{o}}}} \right) + \frac{{1 - \Delta }}{2}\left( {\rho _{{\mathrm{A}}_{\mathrm{e}}} + \rho _{{\mathrm{A}}_{\mathrm{o}}}} \right)} \right],\\ \hskip-4pt g_{\mathrm {MF}}^{{\mathrm{B}}_{\mathrm{e}}{\mathrm{B}}_{\mathrm{o}}} = J_c\left[ {\frac{{1 + \Delta }}{2}\left( {\rho _{{\mathrm{B}}_{\mathrm{e}}} + \rho _{{\mathrm{B}}_{\mathrm{o}}}} \right) - \frac{{1 - \Delta }}{4}\left( {\delta _{{\mathrm{B}}_{\mathrm{e}}} + \delta _{{\mathrm{B}}_{\mathrm{o}}}} \right)} \right],\\ \hskip-4pt g_{\mathrm {MF}}^{{\mathrm{C}}_{\mathrm{e}}{\mathrm{C}}_{\mathrm{o}}} = J_c\left[ {\frac{{1 + \Delta }}{2}\left( {\rho _{{\mathrm{C}}_{\mathrm{e}}} + \rho _{{\mathrm{C}}_{\mathrm{o}}}} \right) - \frac{{1 - \Delta }}{4}\left( {\delta _{{\mathrm{C}}_{\mathrm{e}}} + \delta _{{\mathrm{C}}_{\mathrm{o}}}} \right)} \right],\hskip8pt\end{array}$$

**Fig. 9:**
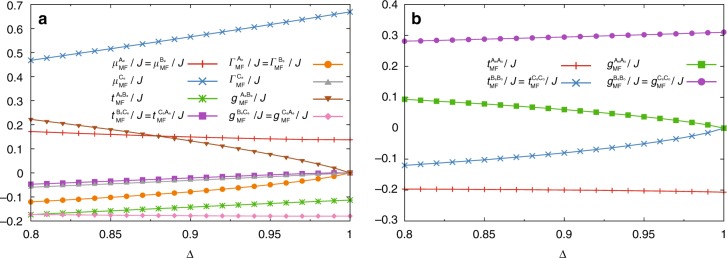
Δ-dependence of the recombined MF parameters. MF parameters associated with (**a**) the intra-layer coupling and (**b**) the inter-layer coupling

Figure 9 shows the Δ-dependence of these MF parameters. Because these MF parameters are real valued, the coefficient matrix in Eq. (20) has the form

25 $$H_{{\mathrm{NLSW}}} = \left( {\begin{array}{*{20}{c}} {P_{\mathbf{k}}} & {Q_{\mathbf{k}}} \\ {Q_{\mathbf{k}}} & {P_{\mathbf{k}}} \end{array}} \right),$$

with

26 $$P_{\mathbf{k}} = \left( {\begin{array}{*{20}{c}} {H_{{\mathrm{ee}},{\mathbf{k}}}} & {H_{{\mathrm{eo}},{\mathbf{k}}}} \\ {H_{{\mathrm{eo}},{\mathbf{k}}}} & {H_{{\mathrm{oo}},{\mathbf{k}}}} \end{array}} \right),Q_{\mathbf{k}} = \left( {\begin{array}{*{20}{c}} {H_{{\mathrm{ee}},{\mathbf{k}}}' } & {H_{{\mathrm{eo}},{\mathbf{k}}}' } \\ {H_{{\mathrm{eo}},{\mathbf{k}}}' } & {H_{{\mathrm{oo}},{\mathbf{k}}}' } \end{array}} \right).$$

This form can be diagonalized by a Bogoliubov transformation,

27 $$\left( {\begin{array}{*{20}{c}} {{\mathbf{a}}_{\mathbf{k}}} \\ {{\bar{\mathbf a}}_{\mathbf{k}}} \\ {{\mathbf{a}}_{ - {\mathbf{k}}}^\dagger } \\ {{\bar{\mathbf a}}_{ - {\mathbf{k}}}^\dagger } \end{array}} \right) = \left( {\begin{array}{*{20}{c}} {U_{\mathbf{k}}} & {V_{\mathbf{k}}} \\ {V_{\mathbf{k}}} & {U_{\mathbf{k}}} \end{array}} \right)\left( {\begin{array}{*{20}{c}} {{\mathbf{\alpha }}_{\mathbf{k}}} \\ {{\mathbf{\alpha }}_{ - {\mathbf{k}}}^\dagger } \end{array}} \right){\kern 1pt} ,$$

where **α**_**k**_ $\left( {{\mathbf{\alpha }}_{ - {\mathbf{k}}}^{\mathrm{\dagger }}} \right)$ is the six-component vector comprising the annihilation (creation) operators of Bogoliubov bosons. The transformation matrices satisfy $U_{\mathbf{k}}^{\mu \kappa } = (U_{ - {\mathbf{k}}}^{\mu \kappa })^ \ast$ and $V_{\mathbf{k}}^{\mu \kappa } = (V_{ - {\mathbf{k}}}^{\mu \kappa })^ \ast$. The poles, *ω*_*κ*,**k**_, are the square-roots of the eigenvalues of *S*^2^(*P*_**k**_ + *Q*_**k**_)(*P*_**k**_−*Q*_**k**_).

When calculating the sublattice magnetization, the reduction of the ordered moment relative to the classical value *S* corresponds to the local magnon density. With the phase factors for each sublattice, $c_{{\mathrm{A}}_{\mathrm{e}}} = c_{{\mathrm{B}}_{\mathrm{e}}} = - c_{{\mathrm{C}}_{\mathrm{e}}} = c_{{\mathrm{A}}_{\mathrm{o}}} = - c_{{\mathrm{B}}_{\mathrm{o}}} = c_{{\mathrm{C}}_{\mathrm{o}}} = 1$ (see Fig. 1a), we have

28 $$\langle S_{\mathbf{r}}^x\rangle = c_\mu \left( {S - \langle a_{\mu ,{\mathbf{r}}}^\dagger a_{\mu ,{\mathbf{r}}}\rangle } \right) = c_\mu \left( {S - \frac{1}{{N_{{\mathrm{mag}}}}}\mathop {\sum}\limits_{\mathbf{k}} \mathop {\sum}\limits_\kappa \left| {V_{\mathbf{k}}^{\mu \kappa }} \right|^2} \right),$$

for site ***r*** in sublattice *μ*.

The dynamical spin structure factor is defined by

29 $$\begin{array}{*{20}{l}} {{\it{{\cal S}}}^{\alpha \alpha }\left( {{\mathbf{q}},\omega } \right)} \hfill & = \hfill & {{\int}_{ - \infty }^\infty \frac{{{\mathrm{d}}t}}{{2\pi }}{\rm e}^{{\rm i}\omega t}\frac{1}{N}\mathop {\sum}\limits_{{\mathbf{r}},{\mathbf{r}}\prime } {\rm e}^{ - {\rm i}{\mathbf{q}} \cdot \left( {{\mathbf{r}} - {\mathbf{r}}\prime } \right)}\left\langle {S_{\mathbf{r}}^\alpha \left( t \right)S_{{\mathbf{r}}\prime }^\alpha \left( 0 \right)} \right\rangle } \hfill \\ {} \hfill & = \hfill & {\mathop {\sum}\limits_n \delta \left( {\omega - \omega _n} \right){\kern 1pt} \left| {\left\langle {0|S_{\mathbf{q}}^\alpha |n} \right\rangle } \right|^2.} \hfill \end{array}$$

where $S_{\mathbf{q}}^\alpha = N^{ - 1/2}\mathop {\sum}\nolimits_{\mathbf{r}} S_{\mathbf{r}}^\alpha {\rm e}^{ - {\rm i}{\mathbf{q}} \cdot {\mathbf{r}}}$ and |*n*〉 and *ω*_*n*_ denote the *n*th excited state and its excitation energy, respectively. The longitudinal spin component is

30 $$S_{\mathbf{q}}^x = \frac{{\sqrt N }}{3}S\left( {\delta _{{\mathbf{q}},0} + \delta _{{\mathbf{q}},{\mathbf{Q}}} + \delta _{{\mathbf{q}}, - {\mathbf{Q}}}} \right) + \delta S_{\mathbf{q}}^x,$$

with **Q** = (1/3, 1/3,1) and

31 $$\delta S_{\mathbf{q}}^x = - \sqrt {\frac{1}{N}} \mathop {\sum}\limits_{\mu ,{\mathbf{k}}} c_\mu a_{\mu ,{\mathbf{k}} - {\mathbf{q}}}^\dagger a_{\mu ,{\mathbf{k}}},$$

We truncate the expansions of the transverse spin components at the lowest order:

32 $$\begin{array}{l}\hskip-4pt S_{\mathbf{q}}^y \approx - {\rm i}\sqrt {\frac{S}{{12}}} \mathop {\sum}\limits_\mu \left( {a_{\mu ,{\mathbf{q}}} - a_{\mu , - {\mathbf{q}}}^\dagger } \right),\hskip13pt\\ S_{\mathbf{q}}^z \approx \sqrt {\frac{S}{{12}}} \mathop {\sum}\limits_\mu \left( { - c_\mu } \right)\left( {a_{\mu ,{\mathbf{q}}} + a_{\mu , - {\mathbf{q}}}^\dagger } \right).\end{array}$$

The transverse components of the dynamical structure factor, ${\it{{\cal S}}}_ \bot \left( {{\mathbf{q}},\omega } \right) = {\it{{\cal S}}}^{yy}\left( {{\mathbf{q}},\omega } \right) + {\it{{\cal S}}}^{zz}\left( {{\mathbf{q}},\omega } \right)$, reveal the magnon dispersion,

33 $$\begin{array}{*{20}{l}} {{\it{{\cal S}}}^{yy}\left( {{\mathbf{q}},\omega } \right)} \hfill & = \hfill & {\mathop {\sum}\limits_n \delta \left( {\omega - \omega _n} \right){\kern 1pt} \left| {\left\langle {0|S_{\mathbf{q}}^y|n} \right\rangle } \right|^2} \hfill \\ {} \hfill & \approx \hfill & {\frac{S}{{12}}\mathop {\sum}\limits_\kappa \delta \left( {\omega - \omega _{\kappa ,{\mathbf{q}}}} \right){\kern 1pt} \left| {\mathop {\sum}\limits_\mu \left( {U_{\mathbf{q}}^{\mu \kappa } - V_{\mathbf{q}}^{\mu \kappa }} \right)} \right|^2,} \hfill \\ {{\it{{\cal S}}}^{zz}\left( {{\mathbf{q}},\omega } \right)} \hfill & = \hfill & {\mathop {\sum}\limits_n \delta \left( {\omega - \omega _n} \right){\kern 1pt} \left| {\langle 0|S_{\mathbf{q}}^z|n\rangle } \right|^2} \hfill \\ {} \hfill & \approx \hfill & {\frac{S}{{12}}\mathop {\sum}\limits_\kappa \delta \left( {\omega - \omega _{\kappa ,{\mathbf{q}}}} \right){\kern 1pt} \left| {\mathop {\sum}\limits_\mu c_\mu \left( {U_{\mathbf{q}}^{\mu \kappa } + V_{\mathbf{q}}^{\mu \kappa }} \right)} \right|^2.} \hfill \end{array}$$

Meanwhile, ${\it{{\cal S}}}^{xx}\left( {{\mathbf{q}},\omega } \right)$ comprises the elastic contribution and the longitudinal fluctuations,

34 $${\it{{\cal S}}}_{||}\left( {{\mathbf{q}},\omega } \right) = \mathop {\sum}\limits_n \delta \left( {\omega - \omega _n} \right){\kern 1pt} \left| {\left\langle {0|\delta S_{\mathbf{q}}^x|n} \right\rangle } \right|^2,$$

which can be evaluated by using Wick’s theorem. The result at *T* = 0 is

35 $${\it{{\cal S}}}_{||}\left( {{\mathbf{q}},\omega } \right) = \Theta \left( \omega \right)N^{ - 1}\mathop {\sum}\limits_{\mathbf{k}} \mathop {\sum}\limits_{\kappa ,\lambda ,\mu ,\nu } c_\mu c_\nu {\mathrm{Re}}{\kern 1pt} A_{\mu \nu ;\kappa \lambda }\left( {{\mathbf{k}};{\mathbf{q}}} \right){\kern 1pt} \delta \left( {\omega - \omega _{\kappa , - {\mathbf{k}} + {\mathbf{q}}} - \omega _{\lambda ,{\mathbf{k}}}} \right),$$

where

36 $${\mathrm{A}}_{\mu \nu ;\kappa \lambda }({\mathbf{k}};{\mathbf{q}}) = \frac{1}{2}\left[ {\left( {U_{{\mathbf{k}} - {\mathbf{q}}}^{\mu \kappa }} \right)^ \ast V_{\mathbf{k}}^{\mu \lambda } + \left( {V_{{\mathbf{k}} - {\mathbf{q}}}^{\mu \kappa }} \right)^ \ast U_{\mathbf{k}}^{\mu \lambda }} \right]{\kern 1pt} \left[ {U_{{\mathbf{k}} - {\mathbf{q}}}^{\nu \kappa }\left( {V_{\mathbf{k}}^{\nu \lambda }} \right)^ \ast + V_{{\mathbf{k}} - {\mathbf{q}}}^{\nu \kappa }\left( {U_{\mathbf{k}}^{\nu \lambda }} \right)^ \ast } \right].$$

### Data availability

All relevant data are available from the corresponding authors upon reasonable request.
