# Author Correction: The nature of spin excitations in the one-third magnetization plateau phase of Ba_3_CoSb_2_O_9_

**DOI:** 10.1038/s41467-018-05679-3

**Published:** 2018-08-01

**Authors:** Y. Kamiya, L. Ge, Tao Hong, Y. Qiu, D. L. Quintero-Castro, Z. Lu, H. B. Cao, M. Matsuda, E. S. Choi, C. D. Batista, M. Mourigal, H. D. Zhou, J. Ma

**Affiliations:** 1 Condensed Matter Theory Laboratory, RIKEN, Wako, 351-0198 Saitama, Japan; 2 School of Physics, Georgia Institute of Technology, 30332 Atlanta, GA USA; 3 Neutron Scattering Division, Oak Ridge National Laboratory, 37831 Oak Ridge, TN USA; 4 NIST Centre for Neutron Research, National Institute of Standards and Technology, 20899 Gaithersburg, MD USA; 5 Helmholtz-Zentrum Berlin für Materialien und Energie, D-14109 Berlin, Germany; 6 National High Magnetic Field Laboratory, Florida State University, 32310 Tallahassee, FL USA; 7 Department of Physics and Astronomy, University of Tennessee, 37996 Knoxville, TN USA; 8 Neutron Scattering Division and Shull-Wollan Center, Oak Ridge National Laboratory, 37831 Oak Ridge, TN USA; 9 Key Laboratory of Artificial Structures and Quantum Control, Department of Physics and Astronomy, Shanghai Jiao Tong University, 200240 Shanghai, China; 10 Collaborative Innovation Center of Advanced Microstructures, 210093 Nanjing, Jiangsu China

Correction to: *Nature Communications*; 10.1038/s41467-018-04914-1; published online 10 July 2018

The original version of this Article omitted the following from the Acknowledgements:

‘J. Ma’s primary affiliation is Shanghai Jiao Tong University.’

This has been corrected in both the PDF and HTML versions of the Article.

